# Mutations in the nucleotide binding pocket of MreB can alter cell curvature and polar morphology in *Caulobacter*

**DOI:** 10.1111/j.1365-2958.2011.07698.x

**Published:** 2011-05-26

**Authors:** Natalie A Dye, Zachary Pincus, Isabelle C Fisher, Lucy Shapiro, Julie A Theriot

**Affiliations:** 1 Department of Biochemistry and Howard Hughes Medical Institute, Stanford UniversityStanford, CA, USA; 2 Department of Microbiology and Immunology, Stanford UniversityStanford, CA, USA; 3 Department of Developmental Biology, Stanford UniversityStanford, CA, USA; 4 Department of Molecular, Cellular, and Developmental Biology, Yale UniversityNew Haven, CT, USA

## Abstract

The maintenance of cell shape in *Caulobacter crescentus* requires the essential gene *mreB*, which encodes a member of the actin superfamily and the target of the antibiotic, A22. We isolated 35 unique A22-resistant *Caulobacter* strains with single amino acid substitutions near the nucleotide binding site of MreB. Mutations that alter cell curvature and mislocalize the intermediate filament crescentin cluster on the back surface of MreB's structure. Another subset have variable cell widths, with wide cell bodies and actively growing thin extensions of the cell poles that concentrate fluorescent MreB. We found that the extent to which MreB localization is perturbed is linearly correlated with the development of pointed cell poles and variable cell widths. Further, we find that a mutation to glycine of two conserved aspartic acid residues that are important for nucleotide hydrolysis in other members of the actin superfamily abolishes robust midcell recruitment of MreB but supports a normal rate of growth. These mutant strains provide novel insight into how MreB's protein structure, subcellular localization, and activity contribute to its function in bacterial cell shape.

## Introduction

The shape of a bacterial cell is determined by the dynamic assembly and destruction of the peptidoglycan cell wall (for reviews, see Holtje, 1998; Cabeen and Jacobs-Wagner, 2005). For rod-shaped cells, growth is thought to occur in two temporally and spatially distinct processes: elongation and division. Recently, cytoplasmic proteins with structural similarity to eukaryotic actin, tubulin and intermediate filaments have been implicated in the maintenance of cell shape (Cabeen and Jacobs-Wagner, 2005; Shih and Rothfield, 2006). The bacterial actin homologue MreB is thought to be involved in the elongation of rod-shaped cells. It is chromosomally encoded in nearly all species that adopt a non-spherical cell shape (Jones *et al*., 2001; Daniel and Errington, 2003). The removal of MreB homologues in the rod-like species *E. coli*, *B. subtilis*, and *Caulobacter* results in wider, spherical or lemon-shaped cells (Jones *et al*., 2001; Figge *et al*., 2004; Gitai *et al*., 2004, Bendezu and de Boer, 2008). This morphological transition involves the mislocalization of enzymes required for peptidoglycan synthesis (Figge *et al*., 2004; Divakaruni *et al*., 2005; 2007; Dye *et al*., 2005; Kawai *et al*., 2009a) and an altered spatial pattern of new wall growth (Daniel and Errington, 2003; Tiyanont *et al*., 2006; Divakaruni *et al*., 2007; Kawai *et al*., 2009b). Bacterial two-hybrid and immunoprecipitation assays have also shown that MreB can physically associate with many of the cytosolic and membrane bound enzymes that synthesize peptidoglycan subunits (Kruse *et al*., 2005; Carballido-Lopez *et al*., 2006; Mohammadi *et al*., 2007; Kawai *et al*., 2009a; White *et al*., 2010). In *E. coli* and *Caulobacter*, the subcellular localization of MreB is cell-cycle regulated (Figge *et al*., 2004; Gitai *et al*., 2004; Vats and Rothfield, 2007), transitioning from an extended helical-like pattern during elongation to a midcell ring prior to division. Taken together, the existing data suggest that MreB is required for the spatial and temporal control of cell wall growth. Nonetheless, the precise molecular mechanism by which MreB contributes to cell shape is unclear.

Members of the actin/Hsp70 superfamily, including MreB, share a common fold comprised of two major symmetric domains linked by a nucleotide binding cleft (Flaherty *et al*., 1991; Bork *et al*., 1992; Kabsch and Holmes, 1995). Common to all of these proteins is the ability to couple ATP hydrolysis with conformational changes that have functional consequences, such as altered binding affinities for partner proteins (Hurley, 1996). For example, numerous F- and G-actin binding proteins have drastically different binding affinities for ATP and ADP-bound actin (Pollard *et al*., 2000). ATP binding, hydrolysis and phosphate release are also known to regulate the dynamic filamentous polymerization of actin (Pollard *et al*., 2000) and ParM (Garner *et al*., 2004; Orlova *et al*., 2007). For the chaperone Hsp70, which is included in the actin superfamily but does not form filamentous polymers like actin or ParM, ATP binding and hydrolysis trigger conformational changes that mediate the interaction between Hsp70 and its substrates (Liberek *et al*., 1991). Thus, nucleotide binding and hydrolysis are conserved regulatory features of this superfamily and likely to also affect MreB function.

Like other members of the actin superfamily, purified MreB binds to and hydrolyses ATP (Esue *et al*., 2005; 2006; Bean and Amann, 2008; Mayer and Amann, 2009). A purified MreB homologue from *Thermatoga maritima* has been shown to polymerize into long filamentous polymers *in vitro* in an ATP-dependent fashion, analogous to purified muscle actin (van den Ent *et al*., 2001; Esue *et al*., 2005; 2006; Bean and Amann, 2008). *B. subtilis* MreB also appears to form polymers *in vitro* but in a way that is independent of nucleotide (Mayer and Amann, 2009); therefore, the exact role that nucleotide hydrolysis plays in self-association and polymer structure remains unclear and may vary by species. Furthermore, filamentous polymers of MreB have not been visualized at high resolution *in vivo* (Swulius *et al*., 2011)*,* so the ultrastructure of the MreB polymer in cells is unknown. Nonetheless, the small molecule A22, which binds to MreB *in vitro* with micromolar affinity, prevents the *in vitro* assembly of long polymers of *T. maritima* MreB (Bean *et al*., 2009). When applied to *Caulobacter* cells, A22 delocalizes MreB and phenocopies the depletion of MreB (Gitai *et al*., 2005). Thus, the ability to self-assemble must be important for function *in vivo*. Furthermore, since A22 is thought to compete with ATP for the same binding site on MreB (Gitai *et al*., 2005; Bean *et al*., 2009), the ability of A22 to disrupt MreB function *in vivo* suggests that the nucleotide cycle is important for regulating MreB function, as it is for other members of the actin superfamily.

In this work, we took a genetic approach to probe the mechanism of MreB in the maintenance of cell shape and the role of the nucleotide cycle in this process. Specifically, we carefully examined a panel of *Caulobacter* strains with spontaneous, viable amino acid substitution mutations in *mreB,* isolated by selecting for resistance to A22. Mutations conferring resistance to A22 arise near the nucleotide binding pocket of MreB (Gitai *et al*., 2005), allowing us to enrich for mutant strains that potentially encode MreB proteins with altered biochemical properties for ATP binding and hydrolysis. While it has been previously noted that A22-resistant strains can have varying morphological phenotypes (Gitai *et al*., 2005; Aaron *et al*., 2007; Charbon *et al*., 2009), no systematic analysis of cell shape in these mutants has been performed. Using quantitative metrics to capture subtle variations in cell shape, we mapped the range of cell shape phenotypes that are influenced by MreB's assembly, subcellular localization and nucleotide cycle.

This work was inspired by a study in which mutations in the gene encoding β-tubulin were isolated in yeast by selecting for resistance to low doses of Benomyl, a microtubule destabilizing drug (Thomas *et al*., 1985). Two of these mutants were actually found to grow faster in a higher concentration of drug or in low temperature (which also destabilizes microtubules). From these data the authors proposed that their mutant strains had microtubules that varied in stability and that the stability of these filaments could be tuned with low concentrations of Benomyl or low temperature. It was later confirmed that these mutants form polymers with altered dynamics *in vivo* and that the presence of Benomyl can further alter microtubule dynamics in these mutants (Dorn *et al*., 2005). Therefore, we thought it possible that different A22-resistant *Caulobacter* would have MreB polymers with varying stabilities and dynamic behaviours in the cell, perhaps tunable by A22. Such mutants could serve as tools for investigating the mechanism of MreB in cellular processes.

We isolated 35 unique substitutions of 25 amino acids of *Caulobacter* MreB. By quantitatively measuring the shapes of cells grown in the presence and absence of A22, we show that changes in cell length, width, curvature and sensitivity to A22 can be partially uncoupled in this collection of mutants. For a subset of the A22-resistant mutants, we show that the subcellular localization of fluorescently labelled MreB is altered. Some of these mutants aberrantly localize MreB to the cell poles and this polar localization is associated with the development of pointed, rather than rounded, cell poles. For at least three mutants, both cell shape and localization of MreB improve in the presence of A22, analogous to the Benomyl-dependent mutations of β–tubulin. The results presented in this work demonstrate that mutations in *mreB* can be used to study the dynamic behaviour of MreB and the resulting consequences for cell shape.

## Results

### A22 enriches for mutations in the nucleotide-binding pocket of MreB in *Caulobacter*

To generate a collection of *mreB* mutant *Caulobacter*, we grew wild-type *Caulobacter* (CB15N) at 30°C on rich media plates containing a modest concentration of A22 (2.5 µg ml^−1^) and selected for spontaneous resistance. We sequenced the *mreB* gene in 89 independently isolated A22-resistant strains. All strains were found to have point mutations in *mreB* (Table S1). We isolated 11 independent strains bearing the T167A mutation, which was also the most common A22-resistant mutant identified by the screen performed previously (Gitai *et al*., 2005). We also obtained multiple isolates of D192G (9), N21S (8), C110S (7) and D16G (6). Nevertheless, saturation was not achieved in this selection, as 27 mutations were isolated only once. Of the 89 strains, we kept only those with a single mutation in *mreB* and could consistently and repeatedly grow well both in the presence and absence of 2.5 µg ml^−1^ A22. We also added a previously isolated strain, Q26P (Aaron *et al*., 2007). Our final collection of viable CB15N-derived, A22-resistant, *mreB* mutant *Caulobacter* strains contains 35 unique substitutions of 25 different amino acids.

**Figure 1:**
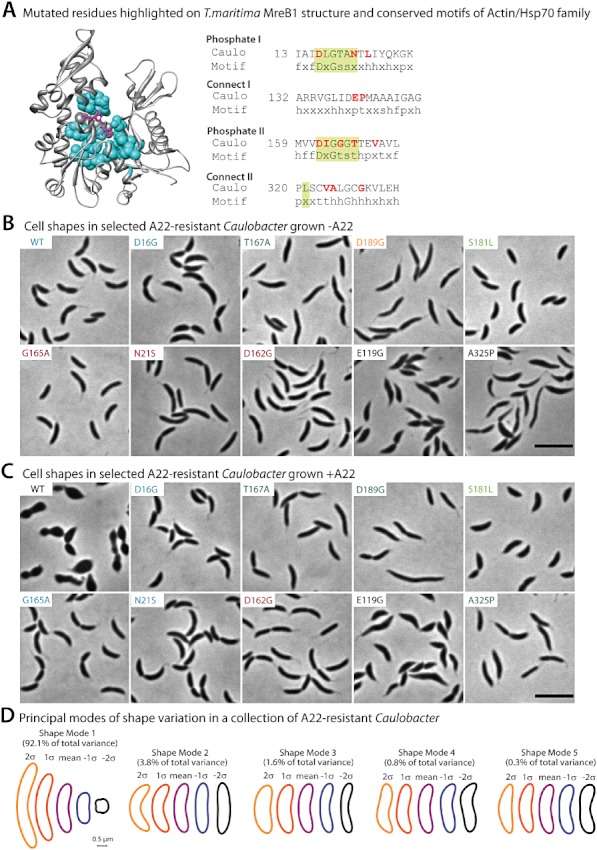
A22-resistant *mreB* mutant *Caulobacter* strains have subtly distinct morphologies. A. Residues conferring A22-resistance highlighted on the backbone crystal structure of *T. maritima* MreB1 (left). The bound nucleotide is purple; mutated residues are shown as space-filling spheres in cyan. On the right, selected mutant residues are highlighted in conserved motifs of the actin/Hsp70 superfamily (Bork *et al*., 1992). The corresponding sequence of *Caulobacter* MreB is presented above each motif. Red text highlights residues that were found to be mutated in this work. Yellow boxes highlight regions that were found to be in close proximity with the nucleotide in the crystal structure of *T. maritima* MreB (van den Ent *et al*., 2001). In the motifs, h = hydrophobic (VLIFWY), f = partly hydrophobic (VLIFWYMCGATKHR), t = tiny (GSAT), s = small (GSATNDVCP), and p = tiny plus polar (GSATNDQEKHR) (Bork *et al*., 1992). Numbers correspond to the first amino acid of each motif in the *Caulobacter* sequence. B and C. Images of representative cells of selected *Caulobacter* strains bearing unique mutations in *mreB* grown in rich media in the absence (B) or presence (C) of 2.5 µg ml^−1^ A22. From left to right, top to bottom, strains are placed in order of the mutated amino acid, with wild-type in the top left corner. Labels are colour coded according to the morphological clustering presented in Fig. 2. Scale bars correspond to 5 µm. D. PCA shape modes derived from the outlines of all cells in the mutant database. The mean shape of all cells is denoted in purple. For each principal component axis, the expected cell shape is shown at one and two standard deviations (σ) from that mean in the direction of that axis.

Based on the crystal structure of *T. maritima* MreB1, the amino acids found to confer A22 resistance in *Caulobacter* primarily reside in the centre of the protein, near the nucleotide binding site (Fig. 1A) (van den Ent *et al*., 2001). This result is consistent with previous work (Gitai *et al*., 2005). There is notoriously little conservation of the primary sequence in the actin/Hsp70 superfamily, including between MreB and actin or ParM. There are, however, short regions of similarity that are preserved throughout the superfamily and are involved in nucleotide binding and conformational flexibility (Bork *et al*., 1992). We aligned the primary sequence of *Caulobacter* MreB to a structure guided alignment of *T. maritima* MreB, yeast actin, and Hsc70 (bovine heat shock cognate protein, 70kD) and found that many of the mutations that we isolated fall within these conserved sequence motifs (Fig. 1A, Fig. S1 and Table S1). Notably, we obtained several isolates of D16G and D162G. These two aspartic acids are conserved across the entire actin superfamily and are predicted to contribute to nucleotide binding and the coordination of the bound metal ion (Flaherty *et al*., 1991; Bork *et al*., 1992; Kabsch and Holmes, 1995; Schuler, 2001), although the biochemical properties of mutant variants have yet to be measured.

We also isolated two strains with mutations at E140. The mutation of the corresponding residue in *E. coli* ParM (E148) has been shown biochemically to abolish ATP hydrolysis *in vitro* and produce a non-functional protein *in vivo* (Garner *et al*., 2004). In actin, as well, the corresponding residue (Q137) is thought to be critically important for ATP hydrolysis (Vorobiev *et al*., 2003; Iwasa *et al*., 2008; Oda *et al*., 2009). Recombinantly purified human cardiac muscle α-actin with Q137 mutated to alanine has a drastically reduced rate of ATP hydrolysis and filament depolymerization (Iwasa *et al*., 2008).

In the conserved sequence motifs of residues involved in the nucleotide cycle, MreB appears to more closely resemble Hsc70 than actin: both MreB and Hsc70 encode acidic residues at positions 140 and 169 (corresponding to 175 and 199 in Hsc70), whereas actin encodes a glutamine and histidine at these respective positions (137 and 161; Fig. S1). In Hsc70, the individual mutations of D10, D175, D199 and D206 to alanine (corresponding to D16, D162, E140 and E169 in *Caulobacter* MreB) all affect the ATP binding and hydrolysis of the purified protein *in vitro* (Wilbanks *et al*., 1994). We isolated substitutions to glycine for three of the four of these key residues in MreB (D16, D162 and E140).

Lastly, we also isolated mutations to amino acids that are not included in the conserved motifs, but their corresponding residues in *T. maritima* MreB are in close proximity with the nucleotide in the crystal structure: D189, D192, E213 and K216 (van den Ent *et al*., 2001).

In conclusion, we propose that the identified mutations, in addition to conferring resistance to A22, are quite likely to affect the ability of MreB to bind and hydrolyse ATP or GTP, even in the absence of drug. Such an alteration in biochemical activity is likely to also affect other aspects of protein function, including self-assembly and interaction with binding partners. Future biochemical experiments will be required to validate these predictions for *Caulobacter* MreB.

### MreB mutants exhibit varying morphologies

To observe how the isolated mutations affect cell growth and shape, we grew all of the strains simultaneously in 96-well format, both with and without A22. Once the cells were steadily growing in log phase, we calculated the doubling time for each strain and rapidly imaged all strains at approximately the same cell density (OD = 0.1–0.25). In each experiment, images of approximately 300 cells per strain, with and without drug (Table S2), were collected.

For this study, we included three types of controls. The first control will be referred to as ‘mutation replicates,’ which are independent isolates of the same mutation. This control was included to monitor potential genetic variability from strain to strain outside of the *mreB* gene. Because we did not mutagenize the parent strain before selecting for A22-resistance, the mutation rate should be low. Thus, we did not anticipate significant differences in the genetic background (outside of *mreB*) that would contribute to the cell shape phenotype. In addition, it has already been confirmed that mutations in *mreB* are responsible for conferring resistance to A22 (Gitai *et al*., 2005). Nonetheless, during the propagation of any strain, mutations can arise. If any aspect of the shape phenotype is caused by mutations outside of *mreB*, there should be variation between the mutation replicates. The 10 most abundant mutations were selected for these mutation replicates. The second control will be referred to as ‘culture replicates’, which are two samples of the same exact strain grown up side-by-side and analysed in parallel. Third, we also performed the entire experiment twice (including all the mutation and culture replicates); we refer to the data sets acquired on different days as ‘experimental replicates’. These last two types of controls allow us to estimate the empirical error and any potential non-genetic variation in cell shape, the former controlling for variation that arises within a single day's data set and the later allowing us to gauge day-to-day variation. In total, this data set contains images of > 55 000 cells in 48 strains (35 unique mutations +13 mutation replicates +2 culture replicates of mutant strains +2 culture replicates of wild-type, all acquired during two experiments performed on different days).

The doubling time of the culture was found to be similar across all mutant strains (Fig. S2), which is perhaps expected, given that we specifically selected only those mutants that could grow well on plates with and without A22. In contrast, variations in cell shape were readily observed (Fig. S3). Images of a subset of the isolated A22-resistant strains grown in the absence or presence of A22 are shown in Fig. 1B and C respectively. The most commonly occurring mutation, T167A, appears straight or sigmoidal, consistent with the previous report (Gitai *et al*., 2005). We also identified other mutations with a similar morphological phenotype (for example D189G and E140G). In addition, we identified many mutant strains that appeared to have a morphology that is close to wild-type (for example, G165A and N21S), much wider than wild-type (E119G), or more variable than wild-type (A325P). Interestingly, different substitutions of the same amino acid have varying effects on cell morphology. For example, A325P exhibits variable cell widths, whereas A325T appears similar to wild-type, though slightly longer (Fig. S3). Importantly, the ‘mutation replicates’ appear to closely resemble each other (Fig. S4), suggesting that it is quite unlikely that any mutations other than the ones in *mreB* could be responsible for these cell shape phenotypes (also discussed in depth below).

To characterize cell shape in an unbiased fashion across our collection of A22-resistant strains, we extracted cell outlines from the phase contrast images and used principal components analysis (PCA) to quantify the primary modes of variation in cell shape for all the cells of our data set (Pincus and Theriot, 2007). The first five modes account for > 98% of the total variation and appear to be biologically meaningful. These modes are illustrated in Fig. 1D. The first mode essentially reflects cell length. It alone accounts for > 92% of the total variation. Since these images were taken from a mixed population of cells in different stages of the cell cycle, and given that cell length will double over the course of the cell cycle, it is not surprising that length accounts for so much of the variation in the data set. Shape mode 2 appears to capture the difference between straight cells and C-shaped cells (referred to hereafter as ‘C-type’ curvature), whereas Shape mode 3 approximates cell width. Shape modes 4 and 5 appear to capture asymmetry within a single cell. High deviations from the mean in Shape mode 4 indicate asymmetry in curvature or the difference between an ‘S-shaped’ cell and a ‘C-shaped’ cell (referred to hereafter as ‘S-type’ curvature). Shape mode 5 appears to reflect variations in width along the long axis of the cell, separating cells with pointed poles from those with rounded poles. Length, average width, and curvature are commonly used metrics of bacterial cell shape and were expected modes of variation, given the known phenotypes of the MreB depletion and the T167A and G165D *mreB* mutants (Figge *et al*., 2004; Gitai *et al*., 2005; Charbon *et al*., 2009). The PCA revealed that asymmetry in curvature and the morphology of the pole are additional informative parameters of shape that are altered in *mreB* mutant strains.

The distributions of values in each of the first five PCA shape modes for each strain grown in the presence or absence of A22 are presented as histograms in Figs S5–9 (corresponding to PCA Shape modes 1–5 respectively). In the following sections, we use this quantitative data set to ask specific questions about the phenotypes of these mutant strains in order to gain insight into MreB's mechanism in cell shape maintenance.

### The morphology of *mreB* mutant strains identifies a putative binding site of A22 on MreB

As mentioned in *Introduction*, we were specifically interested in obtaining A22-dependent mutations, analogous to Benomyl-dependent β-tubulin mutations (Thomas *et al*., 1985). None of the strains exhibited a difference in growth rate in A22, however. In addition, upon visual inspection of the strains (images in Fig. 1B and C and Fig. S3) and the histograms of the shape metrics (Figs S5–9), it is apparent that all of the strains, except wild-type, maintain a fairly rod-like shape in the presence of A22. There are, however, notable differences in cell shape for certain strains grown in the presence and absence of A22. For example, A325P has a largely variable width in the absence of A22 but not in the presence of A22 (Fig. 1B and C and Fig. S7). Other strains do not respond at all or have a much more subtle response to A22 than A325P, suggesting that A22 can affect cell shape in some strains more than others (even though they are all capable of growing at the same rate in A22).

**Figure 2:**
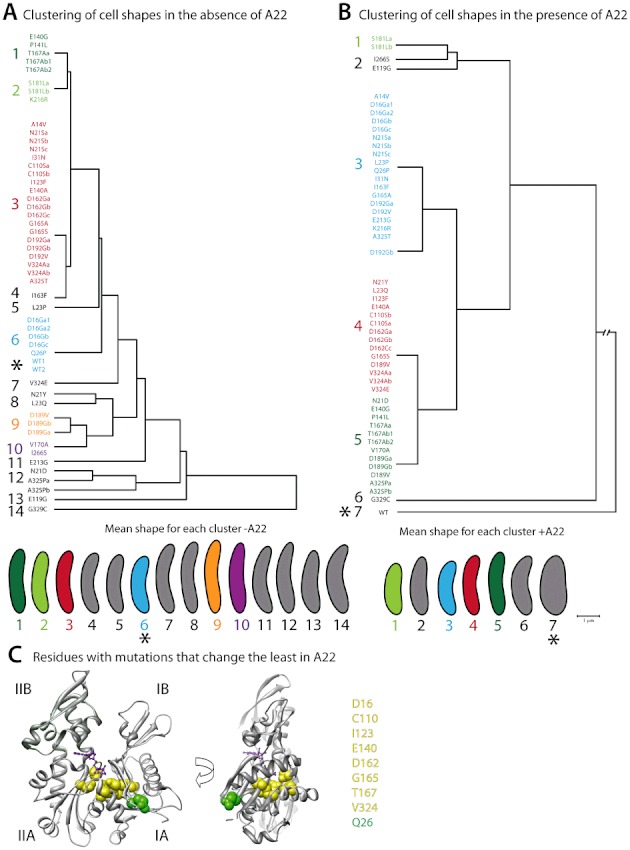
A22 differentially affects the morphology of *mreB* mutant strains. Hierarchical clustering was performed to characterize the cell shape phenotype of strains grown in the absence (A) and presence (B) of A22. Two independent isolates of the same mutation are referred to as ‘mutation replicates’ and are marked here with lowercase letters (a, b, c) after the strain name. For some mutants, we also grew the same exact strain side-by-side and analysed the cell shapes in these two populations separately (referred to as ‘culture replicates’ and marked here with numbers after the strain name). The average amount of day-to-day variation (calculated from the ‘experimental replicates’) was used as a threshold for defining the clusters (see *Experimental procedures*). Variation below this level was considered to be indistinguishable from experimental error. Colours denote the smallest groupings possible that would keep most ‘mutation replicates’ in the same phenotypic class. Asterisks highlight the position of the wild-type samples in both (A) and (B). Note the discontinuity in the line connecting the A22-treated wild-type samples to the resistant strains in part (B), indicating that wild-type cells change shape far more than any of the resistant strains in response to A22. The average cell shape for each grouping (numbered and coloured as in cluster diagrams above) is presented below the clustergrams (drawn to scale). Approximately 300 cells per strain were used to calculate the average shape for each grouping (Table S2). C. The extent to which each strain changes in the presence of A22 was calculated (Fig. S11). Eight out of the nine strains that change the least upon A22 addition (yellow space-filling spheres) cluster together on the crystal structure of *T. maritima* MreB1. Q26 (green space-filling spheres) is the only residue that was not found to cluster at the same site. Bound nucleotide is purple. The subdomains of the structure are labelled according to the convention for the actin/Hsp70 superfamily: I and II correspond to the two large domains of the protein that are linked by the nucleotide-binding cleft, and A and B correspond to the subdomains within these larger domains.

To systematically compare cell shapes with and without A22, we clustered the strains based on the distribution of shapes observed for each strain (Fig. 2A and B). We used the average amount of variation between the two experimental replicates (images captured on different days) to set a threshold for the cluster diagrams. Below this level, any differences in cell shape that exist between two strains cannot be distinguished from those arising from day-to-day variation. All ‘culture replicates’ (marked with numbers in the figures) also cluster below this threshold, indicating that the amount of variation observed between two populations of the same strain is greater if they are grown on different days than if they are grown side-by-side on the same day. Importantly, almost all of the ‘mutation replicates’ (independent isolates of the same mutation, marked with lowercase letters) cluster together in the same group. The only mutation replicates that do not meet this threshold are D189G, A325P and D192G (in A22); even for these strains, however, the mutation replicates cluster near each other and appear by eye to resemble one another (Fig. S4). We believe these strains do not cluster as tightly as the rest because their shape phenotype is inherently more variable (Figs S3, S4 and S10). We conclude that the variation in the *mreB* sequence alone causes the subtle differences in cell shape observed in different clusters.

Interestingly, in the presence of A22, we obtained fewer and tighter clusters than in the absence of A22 (compare Fig. 2A and B), indicating that cell shape in this collection of mutant strains is more variable in the absence of A22. This result was confirmed by directly measuring the shape variance in each strain population (Fig. S10). We measured the difference in cell shape between each strain and the wild-type untreated cell shape and found that in the presence of A22, most strains become more similar to the untreated wild-type cell shape (Fig. S11). However, we found that not all strains have the same magnitude of response to A22 (Fig. 2A and B, Fig. S11). For example, E213G and A325P (black in Fig. 2A) are not clustered tightly with any other strain in the absence of A22 (Fig. 2A), but in the presence of A22, these strains become tightly clustered with many other strains (blue and green groups, respectively, in Fig. 2B). We quantified the extent to which each strain responds to A22 by calculating the difference between the distributions of cell shapes of a given strain in the presence and absence of A22 and confirmed that the magnitude of cell shape change in response to A22 varies depending on the mutation in *mreB* (Fig. S11).

These results demonstrate that A22 can still influence morphology in some of these mutant strains. Note that these strains are all ‘A22-resistant’ in that they can survive and grow well in the presence of A22; nevertheless, the nature of the resistance in each individual strain may vary. To confer A22 resistance, theoretically the mutations in MreB must either considerably lower the affinity for A22 or alter the consequence of A22 binding for MreB function. For example, a mutation that stabilizes MreB polymers could make it more resistant to the destabilizing inhibitor A22 (Gitai *et al*., 2005; Bean *et al*., 2009), in the same way that certain β-tubulin mutations were originally thought to both alter microtubule dynamics and confer resistance to the destabilizing drug, Benomyl (Thomas *et al*., 1985).

In Fig. 2C, we highlight nine amino acids (∼ 25% of the collection) that when mutated produce cells that change the least with A22 (D16, Q26, C110, I123, E140, D162, G165, T167 and V324). Eight of these residues cluster together on the protein's structure near the terminal phosphate of the bound nucleotide (Fig. 2C). We predict that these residues contribute to the binding site of A22 and that the mutation of these residues greatly decreases the affinity of A22 for MreB. Indeed this site is consistent with that identified by *in silico* molecular docking experiments (Bean *et al*., 2009). The residue Q26 is the only amino acid of this subset that does not cluster with the others at this site. We think it is likely that the mutation of this residue to proline (Q26P) has long-range effects on the protein structure.

The nine strains that exhibit the least response to A22 do not group together in the morphological clustering presented in Fig. 2A and B. Thus, a substitution at any one of these amino acids is likely to hinder A22 binding, but the consequences for cell shape in the absence of drug depend on the specific amino acid substitution. The remaining mutations may alter MreB's structure, conformation or function, without considerably altering the protein's affinity for A22. The quantitative analysis presented here both predicts a putative A22 binding site and highlights two strains (A325P and E213G) that are considerably more A22-responsive than the others with respect to cell shape.

### Curvature and width phenotypes of *mreB* mutants are separable

In addition to its well-known role in cell width maintenance, *Caulobacter* MreB has also been implicated in the maintenance of cell curvature. Previous work suggests that MreB affects curvature through crescentin, a putative intermediate filament homologue that is required for cell curvature in *Caulobacter* (Ausmees *et al*., 2003). MreB and crescentin can be co-immunoprecipitated from cell lysates, indicating that the two proteins can physically associate (directly or indirectly) in the same complex (Charbon *et al*., 2009). The treatment of *Caulobacter* with A22 disrupts crescentin localization, causing the crescentin to detach from the cell surface and recoil into a loose helix (Charbon *et al*., 2009), suggesting that MreB is required for proper attachment of crescentin to the cell envelope. Lastly, it has been previously shown that A22-resistant strains carrying the MreB mutations T167A or G165D are straighter, in addition to being longer and thinner, than wild-type (Gitai *et al*., 2005; Charbon *et al*., 2009). For G165D, this defect in curvature was shown to be associated with aberrant crescentin localization (Charbon *et al*., 2009).

**Figure 3:**
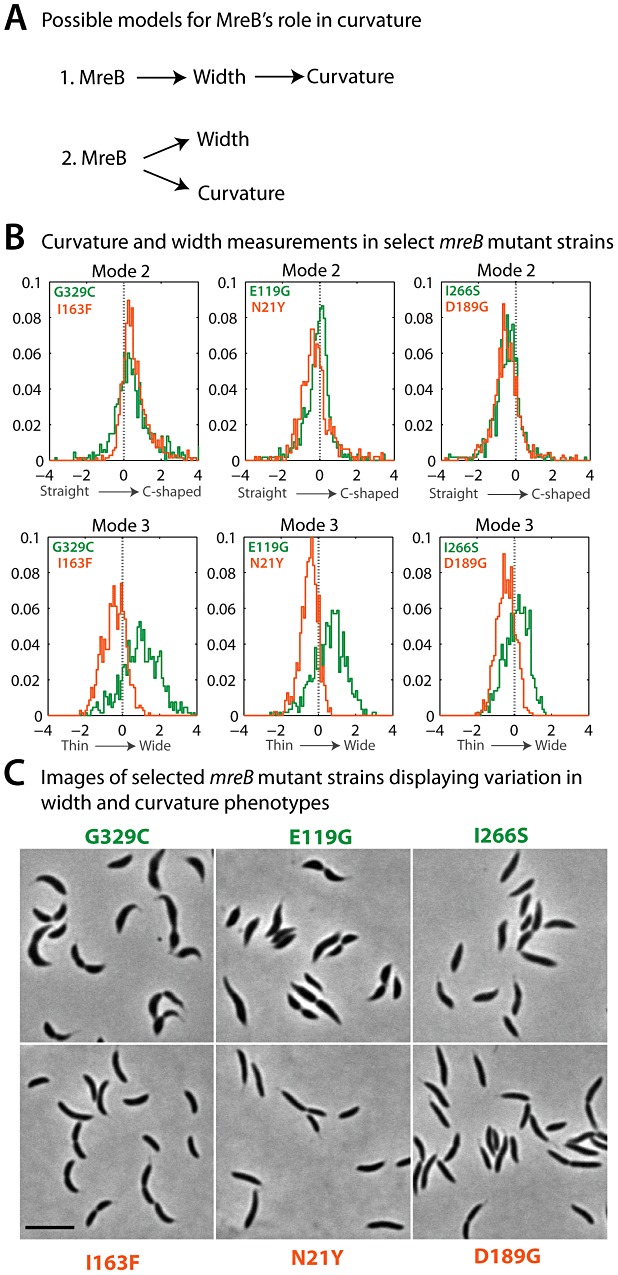
Curvature and width phenotypes are separable in *mreB* mutants. A. Illustration of the two possible pathways by which MreB could affect both width and curvature. In the first, MreB affects curvature via width in the same pathway. In the second, MreB affects curvature and width via distinct and separable pathways. B. Six mutant strains were selected to demonstrate the lack of correlation between curvature and width phenotypes in the collection of *mreB* mutant strains. Shown are histograms of the values in Shape mode 2 (top), corresponding to C-shaped curvature, and Shape mode 3 (bottom), corresponding to cell width, for each pair of strains. For each pair, strains have almost identical distributions in Shape mode 2 but different distributions in Shape mode 3. C. Representative images of the strains analysed in (B). Strains are organized from left to right in order of decreasing Shape mode 2 values and from top to bottom in order of decreasing Shape mode 3 values. Scale bar represents 5 µm.

It is not known how MreB's functions in cell width and curvature are related. It is possible that the crescentin association and the curvature phenotype are downstream of the width-determination pathway, such that any perturbations in cell width are coupled to defects in cell curvature (Fig. 3A). Because deletion of crescentin does not affect cell width, curvature cannot be upstream of cell width in the same pathway (Ausmees *et al*., 2003). Alternatively, it is possible that the pathways determining cell width and curvature are distinct. If the latter, it should be possible to isolate mutations that perturb one pathway but not the other. To distinguish between these two possible models, we looked for coupling between the curvature and width phenotypes in our collection of *mreB* mutants.

In our large collection of *mreB* mutant morphologies, defects in length, width and curvature appear to be described by distinct PCA Shape modes (Fig. 1D). By construction, the PCA Shape modes are orthogonal deviations from the mean shape of all cells (regardless of their genotype or cell cycle stage) and are thus uncorrelated with one another across the entire data set of individual cells. However, different strains occupy different regions of the PCA ‘shape space’ (not shown), and thus it is reasonable to ask whether these parameters, averaged per strain, are correlated among different strains. Overall, we find that PCA Shape modes 1–5, corresponding roughly to length, C-type curvature, width, S-type curvature, and pole shape, are not tightly correlated (Fig. S12). The highest correlation was observed between Shape mode 1 and Shape mode 4 (*R* = 0.67, *P* < 10^−10^), indicating that strains that are longer than average are also likely to be S-shaped. A modest but statistically significant correlation was also observed between Shape mode 1 with Shape mode 2 (*R* = −0.52, *P* < 0.0002) and Shape mode 1 with Shape mode 3 (*R* = −0.59, *P* < 0.0002), indicating that changes in length are also mildly associated with changes in width and curvature. Importantly, Shape mode 3, which approximates cell width, is not correlated with either Shape mode 2 or Shape mode 4, suggesting that changes in cell width are not coupled with either of the two modes approximating cell curvature.

As a further test, we directly measured the length and average width of each bacterium along and about its centerline (respectively) and devised *ad hoc* metrics to capture overall curvature (C-type + S-type), shape asymmetry and the ratio of width at the middle to that at the poles (see *Experimental procedures*). These metrics are also not tightly correlated with one another (Fig. S12), with the exception of cell length and asymmetry. Thus, strains that are longer on average tend to also be more asymmetric and S-shaped. As it is redundant with cell length when comparing strains, we did not use Shape mode 4 or asymmetry in subsequent analyses.

The low degree of correlation between the multiple metrics of curvature and cell width in our data set of mean shapes for each strain suggests that these two phenotypes can be uncoupled with *mreB* mutations and that MreB affects cell width and curvature via distinct pathways (model 2 in Fig. 3A). We confirmed this separation of shape phenotypes by selecting a few example strains that have the same average value for Shape mode 2 but different average values for Shape mode 3 (Fig. 3B and C). In these three pairs of examples, it is evident from the distributions of values in Shape modes 2 and 3 (Fig. 3B) and in the images of the cells (Fig. 3C) that cell width can vary without coordinated alterations in cell curvature. We conclude that MreB affects cell width and curvature via distinct pathways and probably binding partners, and that these pathways can be genetically uncoupled.

### Morphological analysis identifies a putative binding site on MreB for curvature-mediating factors

**Figure 4:**
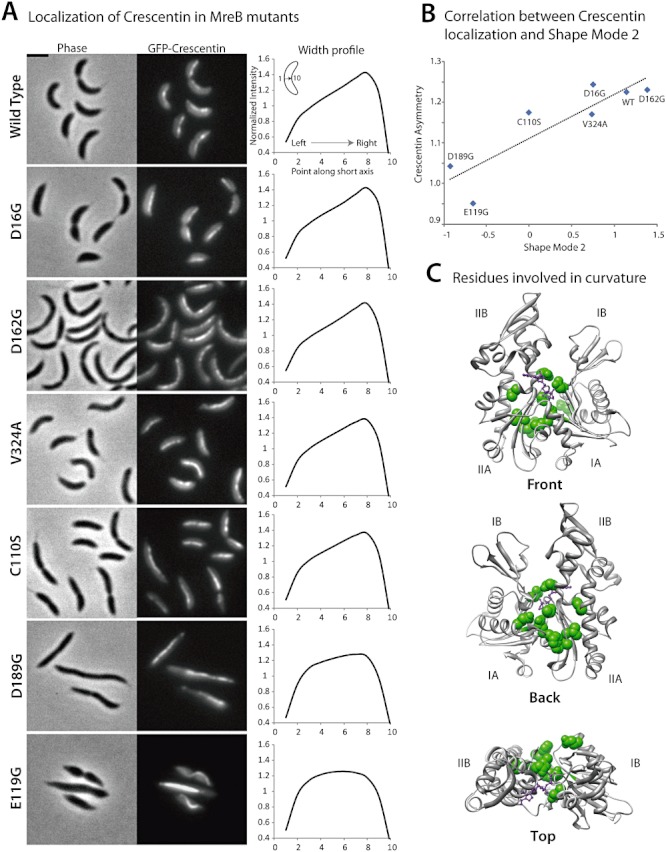
Curvature and crescentin localization are disrupted in a subset of *mreB* mutants. A. GFP–crescentin localization in selected MreB mutant strains grown in the absence of A22. GFP–crescentin was expressed from its native promoter in the presence of unlabelled wild-type crescentin in the A22-resistant MreB mutant strains. Representative cells are shown in phase contrast (left) and GFP fluorescence (centre). On the right is the average one-dimensional intensity of crescentin along the short axis of the cell in a mixed population of each strain. To generate this plot, the GFP intensity was averaged along a line running from pole to pole in each cell at ten equally spaced points along the cell width (see *Experimental procedures*). Total intensities were normalized, so that the measured variation from cell to cell is only in the distribution and not in abundance. At least 300 cells per strain were averaged. Strains are in order (from top to bottom) of increasing crescentin mislocalization. B. The degree of crescentin asymmetry along the short axis of the cell was measured as the ratio of intensity on the right side of the cell to that in the centre of the cell. This value correlates with the average Shape mode 2 value for each strain. C. Residues that when mutated produce cells that are straighter than wild-type (N21, C110, E119, E140, P141, T167, V170, S181, D189 and I266) are highlighted in green space-filling spheres on the crystal structure of *T. maritima* MreB1 visualized from three orientations. Note that N21 was included because the mutation of this residue to Y or D produces cells with aberrant curvature, but the mutation of this residue to S does not have the same effect. Likewise, E140G was far less curved than E140A. Bound nucleotide is purple. The subdomains of the structure are labelled according to the convention for the actin/Hsp70 superfamily: I and II correspond to the two large domains of the protein that are linked by the nucleotide-binding cleft, and A and B correspond to the subdomains within these larger domains.

Given the established connection between MreB and crescentin and the results discussed above, we hypothesized that the subset of our MreB mutant strains that exhibit abnormal cell curvature might have altered interactions between MreB and crescentin. Therefore, we determined the localization of GFP–crescentin in selected mutant MreB strains that would represent the range of the cell shape phenotypes (Fig. 4A).

As shown previously, GFP–crescentin follows the inner curvature of wild-type cells (Ausmees *et al*., 2003). In these mutant strains, GFP–crescentin localization is disrupted to varying degrees. In strains bearing the V324A or C110S mutation, crescentin localization is mostly normal but occasionally appears to run diagonal to the long axis of the cell or lie in the middle of the cell, rather than at the cell edge. In D189G and E119G (which are notably straighter than wild-type), irregularities became more common. In some cells, particularly in the E119G strain, the localization of crescentin resembles that in A22-treated cells, appearing helical and ‘detached’ from the cell membrane. To quantify this phenotype, we measured the distribution of GFP–crescentin along the short axis of the cell (see *Experimental procedures*). GFP intensity was averaged from pole to pole and plotted as a function of the position along the cell width axis (Fig. 4A, right). This width profile is asymmetric for the wild-type strain, indicating that GFP–crescentin is peaked on the side of inner curvature (cells were aligned with this side to the right). This profile becomes symmetric for a strain that contains cells that have crescentin running diagonal to the long axis of the cell, parallel to the long axis but in the middle of the cell (rather than at the cell edge), or completely detached from the membrane in a loose helix. The extent of asymmetry was calculated as the ratio of the intensity of GFP–crescentin on the right side of the cell to that in the middle of the cell. We found that crescentin asymmetry correlates closely with Shape mode 2 (Fig. 4B, *R* = 0.88, *P* = 0.01), indicating that strains that mislocalize crescentin are straighter.

From these data, we conclude that crescentin is mislocalized in some but not all of the A22-resistant strains and that the curvature phenotype can be correlated with proper localization of the intermediate filament homologue crescentin. In Fig. 4C, we highlight the 10 residues with the lowest Shape mode 2 values (straighter than WT) on the crystal structure of *T. maritima* MreB. Remarkably, these residues appear to reside in a cluster on the back of the protein, perhaps reflecting a binding site for either crescentin or another protein mediating an interaction between crescentin and MreB. Alternatively, these residues may affect the ability of MreB to self-associate or interact with the cell envelope in a way that specifically but indirectly perturbs curvature and crescentin localization.

### A subset of mutants exhibit highly variable cell widths

**Figure 5:**
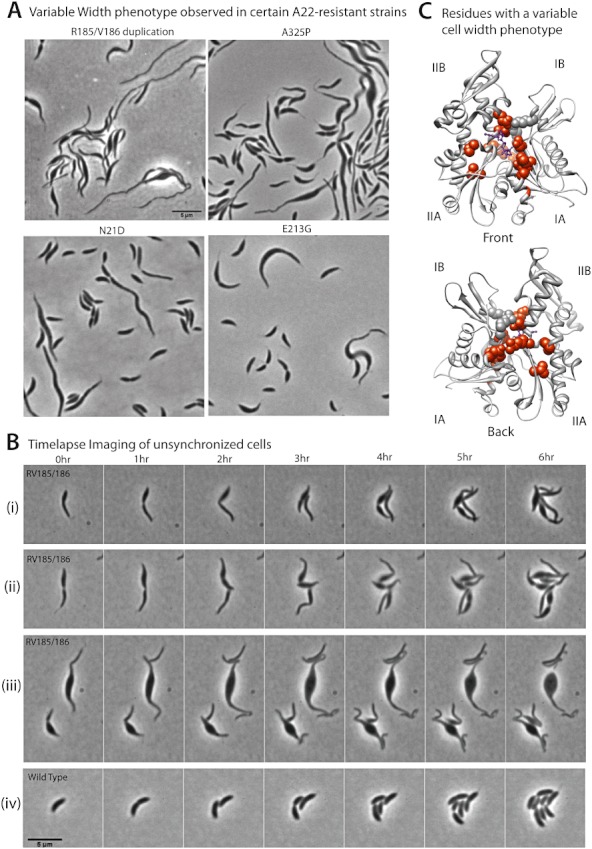
Some A22-resistant strains exhibit a variable width phenotype. A. Static images of mixed populations of strains that do not tightly control their cell width. B. Time-lapse imaging of unsynchronized populations of the R185V186 duplication strain on a PYE + 1% agarose pad without A22. (i), (ii) and (iii) are separate cells imaged simultaneously on the same slide. (iv) Wild-type cells grown under the same conditions as those in (Bi–iii). C. Residues that when mutated produce the largest deviations in Shape mode 5 (producing pointed poles) are highlighted on the crystal structure of *T. maritima* MreB in orange: D16, L23, E119, V170, R185/V186, D189, I266, E213, A325 and G329. In grey, residues that appear to make inter-domain contacts with residues that were mutated in this work (K58 and R75). Bound nucleotide is purple.

In this work, we have identified several mutants with perturbations in cell width, including strains that are thinner, wider, or more variable in cell width than wild-type. While mutations that produce thinner cells have been previously identified (Gitai *et al*., 2005; Charbon *et al*., 2009), and wider cells are known to emerge from the loss of MreB function (Figge *et al*., 2004; Gitai *et al*., 2004; 2005), mutations that increase the variability in cell width within a single cell have not yet been described in *Caulobacter*. Given that the shape of wild-type cells is fairly constant within a population, we were particularly interested in the strains that seemed to have a more variable cell shape, including A325P and, to a lesser extent, N21D and E213G (Fig. 5A, Fig. S7). In these strains, it is possible to see large deviations in cell width across the population and even within single cells. Many cells have long, thin extensions near the poles and wide cell centres.

In addition to the strains listed above, we also isolated a mutant strain with an extreme version of this variable width phenotype (Fig. 5A). The nature of the mutation in this strain is also unique, as it is a small duplication: R185 and V186 are repeated, so that the sequence VRV at amino acids 184–186 becomes VRVRV. While we had isolated this mutation in the wild-type *Caulobacter* strain in our initial screen (not shown), the phenotype was rapidly suppressed and it was removed from our collection. Later, upon sequencing spontaneous A22-resistant mutants of a GFP–MreB merodiploid strain, which contains the endogenous, unlabelled *mreB*, as well as a xylose-inducible GFP–MreB, we isolated the same mutation with the same dramatic morphological phenotype (Fig. 5A). This mutation is present only in the endogenous unlabelled copy of *mreB*; the copy of *mreB* encoded at the x*ylX* locus has a wild-type sequence (data not shown). In this strain, the phenotype is relatively stable, with or without the addition of xylose to induce the wild-type GFP–MreB (data not shown). It is likely that a low level of the wild-type *gfp–mreB* is expressed without induction and prevents this mutation from being quickly lost from the population. Still, if grown for long periods of time without A22, this strain will also yield suppressors.

Long, thin projections have also been observed in a strain of *Caulobacter* containing a mutation in *ftsZ*, the tubulin homologue required for division (Wang *et al*., 2001; Li *et al*., 2007). This mutation causes a severe late block in division that leaves two cells attached by a thin bridge resulting from a failed division event. We wondered whether the thin regions of these *mreB* mutant cells could also be derived from failed division events. To answer this question, we used time-lapse microscopy to image the R185V186 duplication strain over multiple rounds of growth and division (Fig. 5B and Movies S1–3). In these movies, it is apparent that these polar extensions are actively elongated throughout the cell cycle and do not derive from failed division events. In addition, we can see that extremely thin cells can be pinched off from the ends of these extensions and go on to grow and divide again. We observed several thin cells elongate and divide relatively symmetrically to produce two thin daughter cells (Fig. 5B, i, Movie S1). We also observed cells with an asymmetric cell width, with one end slightly thinner than the other (Fig. 5B, ii, Movie S2). In this case, division produces one thin cell and one slightly wider cell. In the next round of division, this wide cell either grows and divides symmetrically into two wide cells (often with polar extensions), or asymmetrically to produce one thin cell and one wide cell (Fig. 5B, ii, Movie S3). At some point, the cells become too wide to divide. These cells appear to be a terminal state that can only elongate at the ends and eventually lyse (Fig. 5B, iii). This pattern of growth and division is markedly different from that of wild-type *Caulobacter* (Fig. 5B, iv and Movie S4).

This variable width phenotype is best described by PCA Shape mode 5, which distinguishes cells based on the shape of the pole (Fig. 1D). Shape mode 5 values are also well correlated with the standard deviation in Shape mode 3 and variation in average cell width (data not shown). In Fig. 4C, we highlight the ten residues that when mutated produce cells with the largest deviations in Shape mode 5 on the crystal structure of *T. maritima* MreB1. Unlike those residues involved in curvature, the residues implicated in this variable width phenotype are more dispersed, although they seem to reside in the interfaces between the subdomains of the protein structure. Thus, these residues could have an important role in the nucleotide-dependent conformational change of MreB (see *Discussion*).

### Mutations in MreB affect its subcellular localization

To generate the observed cell shape phenotypes, the mutations in MreB could alter the subcellular localization, activity or ultrastructure of MreB polymers. While the precise molecular activity and ultrastructure of *Caulobacter* MreB is unknown, its subcellular localization has been well-characterized (Figge *et al*., 2004; Gitai *et al*., 2004). Thus, we examined the subcellular localization of selected MreB mutants that were chosen to represent the range of morphological classes.

We constructed N-terminal fusions of the mutant MreB proteins to Venus, a yellow-emitting derivative of GFP (Hsu *et al*., 2009), and inserted them into the chromosome at the *xylX* site. The resulting strains each contain two copies of *mreB*: one inducible fluorescently tagged version and one endogenously expressed untagged version. When inserted into a wild-type background, the resulting strain carries wild-type *mreB* at its endogenous locus and the mutant Venus-fusion at the xylose-inducible site. In all cases, the induction of the Venus-mutant MreB fusion was found to confer resistance to A22 (Fig. S13), indicating that the Venus-fusions are functional and that they are dominant to wild-type with respect to growth in A22.

**Figure 6:**
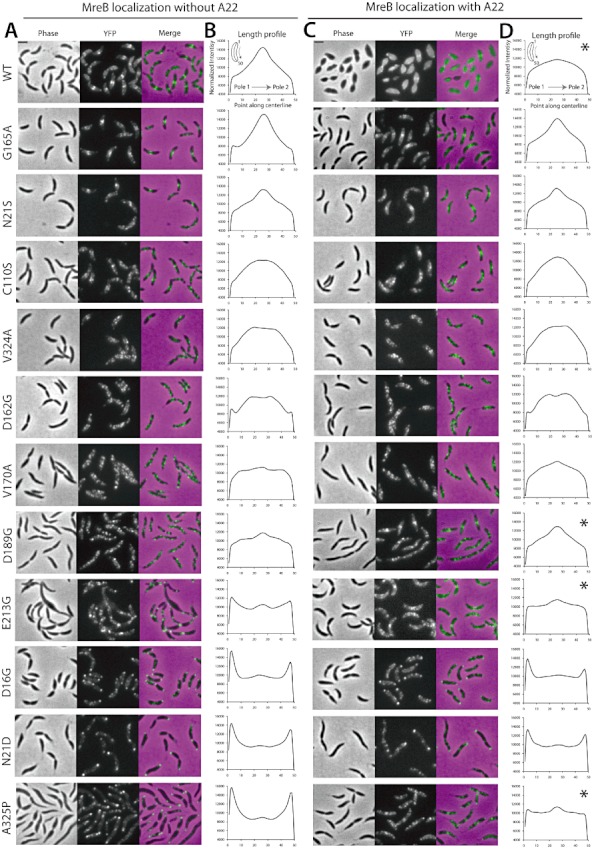
Some mutations in MreB alter its subcellular distribution. A. Representative cells from wild-type and 11 A22-resistant *mreB* mutant strains expressing Venus–MreB in the absence of A22. Images, from left to right, correspond to phase contrast, YFP fluorescence, and a merge of the two (Venus–MreB in green; phase contrast in magenta). B. Average one-dimensional intensity of MreB along the long axis of the cell in a mixed population of each strain. To generate this plot, the fluorescence intensity was averaged along the entire cell width at 50 equally spaced points along the cell length (see *Experimental procedures*). Total intensities were normalized, so that the measured variation from cell to cell is only in the distribution and not in abundance of MreB. At least 500 cells per strain were averaged. C and D. As in (A) and (B) for strains growing in the presence of A22. Asterisks highlight strains in which MreB distribution changes substantially in the presence of A22. All mutant strains shown have the genotype *P_mreB_::mreB^mutant^* + *P_xyl_::venus–mreB^mutant^* (no wild-type protein is present in any of the mutant strains). Scale bars represent 2 µm.

To visualize the subcellular distribution of each mutant protein, we moved the xylose-inducible Venus fusion construct from the wild-type background into the mutant background. The resulting strains contain two matching mutant copies of *mreB* (one fused to Venus), with no wild-type MreB present. We induced the expression of the Venus–MreB^mutant^ fusion (in this background of unlabelled mutant protein) with 0.03% xylose for 1–1.5 h. Representative images of unsynchronized populations of cells grown in the absence of A22 are shown in Fig. 6A.

Wild-type *Caulobacter* MreB transitions between ring and helical patterns over the course of the cell cycle (Figge *et al*., 2004; Gitai *et al*., 2004). It has been previously shown that the A22-resistance mutations T167A, Q26P and G165D abolish the cell cycle regulated transition of MreB from a lengthwise helix along the cell sides to a midcell ring at the division plane (Gitai *et al*., 2005; Aaron *et al*., 2007; Charbon *et al*., 2009). These MreB mutants are dispersed in a lengthwise helical-like pattern throughout the cell cycle. We observed a similar pattern for the mutants V324A, D162G, V170A and D189G (Fig. 6A, middle). In contrast, the mutants G165A, N21S and C110S appear to partially localize to the midcell region (Fig. 6A, top), whereas the mutants E213G, D16G, N21D and A325P localize to the poles of the cell (Fig. 6A, bottom).

From these data, we reasoned that the distribution of MreB into different subcellular regions – the cylindrical side walls, midcell and the poles – could be a distinguishing characteristic of these strains and have important consequences for the cell shape phenotype. In Fig. 6B, we present the average one-dimensional intensity profile of Venus–MreB along the centerline of all cells in a given strain, regardless of cell cycle stage. In wild-type, even with a mixed population of cells, the average one-dimensional intensity of MreB along the centerline is peaked at midcell. The profiles for the A22-resistant MreB proteins appear to lie on a continuum from peaked at midcell, to broadly distributed about midcell and away from the poles, to uniformly distributed, to polar. Note that the mutations D16G and D162G, which are in residues that are conserved throughout nearly all members of the actin superfamily and are thought to play important roles in ATP hydrolysis (see above), do not have identical localization patterns but neither are able to robustly localize to midcell.

**Figure 7:**
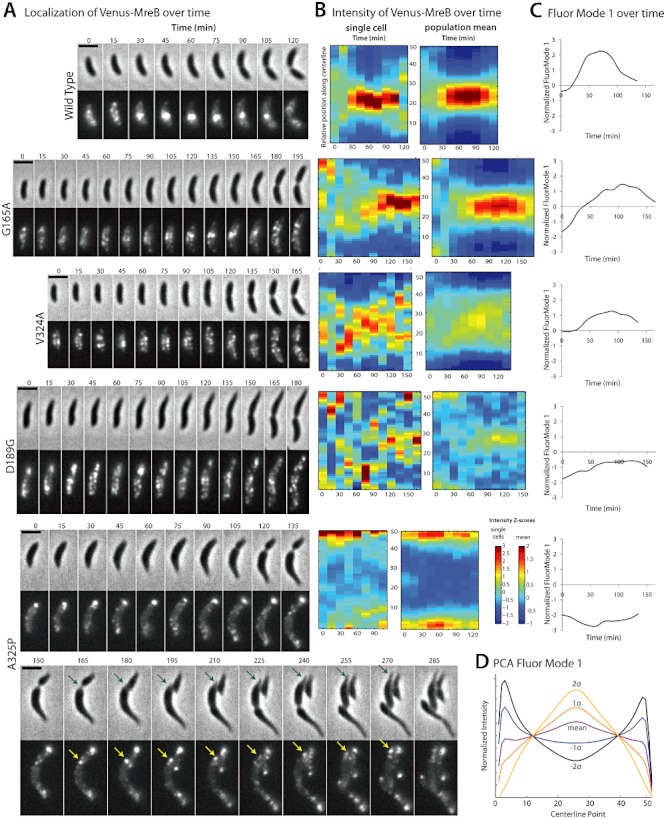
Some mutations in MreB alter its cell cycle-regulated localization pattern. A. Images of representative cells of strains bearing xylose-inducible Venus fusions to wild-type, G165A, V324A, D189G or A325P variants of MreB. Cells were synchronized and imaged with phase contrast (top) and fluorescence (bottom) while growing at room temperature on PYE + 1% agarose. Scale bars represent 2 µm. The time elapsed since the start of the experiment is noted (in minutes) on top of each image. The arrows in the A325P time-lapse highlight the localization of MreB (yellow arrows) in the thin polar extensions visible by phase (green arrows). B. Intensity of Venus–MreB in relative coordinates (normalized to a constant cell length) along the centerline (long axis of the cell) over time (minutes). Intensity profiles were normalized over the entire time-lapse sequence and are presented as a heat map, where red is high and blue is low. On the left is the data that corresponds to the images shown in (A). On the right is the population mean for each strain. Intensity profiles plotted as a function of absolute, rather than relative, cell length are included in Fig. S15. For wild-type, G165A, V324A, D189G, A325P, *n* = 104, 83, 80, 42 and 85 respectively. C. PCA Fluor mode 1, which captures the variation between MreB peaked at the poles (negative values) versus peaked at midcell (positive values) (see part D), is plotted as a function of time for the population averages shown in (B). D. Principal components analysis was performed on the normalized fluorescent MreB distributions of all analysed mutants, and the primary mode of variation is shown (Fluor mode 1). The normalized mean profile of all cells (calculated using all strains shown in Fig. 6) is denoted in purple. Profiles that represent one or two standard deviations (σ) from that mean along the first principal component axis (Fluor mode 1) in each direction are also shown. More information on the calculation of Fluor mode 1 is included in the *Experimental procedures* section and Fig. S14. As in Fig. 6, all mutant strains shown have the genotype *P_mreB_::mreB^mutant^* + *P_xyl_::venus–mreB^mutant^* (no wild-type protein is present in any of the mutant strains).

To discover meaningful parameters for describing the intensity profile of MreB along the centerline in each cell in an unbiased fashion, we again used PCA, this time to assess the principal modes of variation in the distribution of MreB along the centerline (rather than in cell shape). We found that the primary mode of variation in the data set containing all cell intensity profiles (hereafter referred to as ‘Fluor mode 1’) appears to capture the difference between peaked, flat, and polar distributions (Fig. 7D, Fig. S14). Thus, we used this metric to quantify the localization of MreB in these strains.

Given that wild-type MreB delocalizes with A22 treatment, it seems plausible that one way in which A22 could affect morphology in these mutant strains is by affecting the subcellular localization of MreB. To characterize the effect that A22 has on the localization of our mutant MreB proteins, we grew the strains for an extended period of time in the presence of A22 and then induced the expression of the fluorescent variants for 1 h prior to imaging. Representative images and measurements of the intensity of MreB along the centerline are shown in Fig. 6C and D. In the presence of A22, wild-type MreB delocalizes from the membrane and uniformly fills the cytoplasmic volume. In contrast, all of the mutant strains that were analysed maintained a punctate localization at the membrane in A22. Nonetheless, the distribution of this localization throughout the cell changes in some but not all of the strains in the presence of A22. Specifically, E213G and A325P are largely polar in the absence of A22 (Fig. 6A and B) but are much more uniform or slightly peaked at midcell in the presence of A22 (Fig. 6C and D). In contrast, the localizations of D16G and N21D mutants do not change as much in the presence of A22. Even though Venus–MreB is polar in all four of these strains, only two strains respond to A22. Thus, the ability to respond to A22 is not correlated with any particular morphological or localization phenotype.

### The localization of MreB mutants can be uncoupled from the cell cycle

Given that wild-type MreB changes its subcellular localization over the course of the cell cycle, we reasoned that much of the variation in Fluor mode 1 in each strain is likely to be due to cell cycle variation. To directly measure the localization pattern of these mutant MreB proteins over time, we performed time-lapse imaging of Venus–MreB in synchronized populations of selected strains. For each cell, we calculated the cell length, the intensity of MreB along the centerline, and the PCA Fluor mode 1 (Fig. 7D) describing this profile. One representative cell for each strain is shown in Fig. 7A and Movies S5–9. The intensity of MreB along the centerline over time for that individual cell and for the population mean is shown in Fig. 7B. The mean values of Fluor mode 1 at each time point are presented for each strain in Fig. 7C.

As expected from previous data (Figge *et al*., 2004; Gitai *et al*., 2004), the wild-type protein was found to localize to a tight band, offset slightly from midcell, approximately 30 min after the start of imaging. It remains in a tight band, at a constant relative position in the cell, until just before division. Fluor mode 1 is slightly negative at the beginning of the experiment, corresponding to a slightly polar localization of MreB. This value peaks around 60 min, corresponding to a midcell accumulation of MreB, and then drops again before division, reflecting the relocalization of MreB to the cell sides. The strain bearing Venus–MreB^G165A^ has a similar pattern of localization through the cell cycle, although it appears that the entire cell cycle is slightly delayed. The average time to division in this strain was also found to be slightly longer (data not shown). Fluor mode 1 in this strain, as in WT, is negative at the beginning of the cell cycle, corresponding to polar MreB localization. This value gradually increases as the cell slowly accumulates a peak of MreB near midcell. This midcell peak never quite becomes as tight or persists for as long as those in wild-type cells. Nonetheless, it appears that this strain is able to at least partially regulate the localization of MreB as a function of the cell cycle. In contrast, the V324A and D189G variants do not exhibit a wild-type cell cycle localization pattern. The patterns for Venus–MreB^V324A^ and Venus–MreB^D189G^ are subtly, but consistently, different. While neither is recruited to a tight midcell band, Venus–MreB^V324A^ avoids the poles, whereas Venus–MreB^D189G^ can localize along the entire cell length. This difference is apparent both in the plots of intensity along the centerline over time (Fig. 7B) and in the plots of the Fluor mode 1 over time (Fig. 7C). The subcellular localization of Venus–MreB^A325P^ is also not strongly cell cycle regulated: it remains at or near the pole throughout (Fig. 7C, bottom). Note that in these cells, the localization of Venus–MreB^A325P^ is associated with the thinnest parts of the cells (Fig. 7A, bottom, arrows). Upon division, the new pole quickly acquires a new polar spot of MreB (*t* = 165 min, arrow). This polar spot then remains associated with the pole throughout the next cell cycle, as the pole gradually grows into a thin polar extension. Future work will address the molecular requirements for this polar accumulation of MreB, as well as the consequences for polar development.

### The localization of MreB to the cell pole correlates with pointed poles

**Figure 8:**
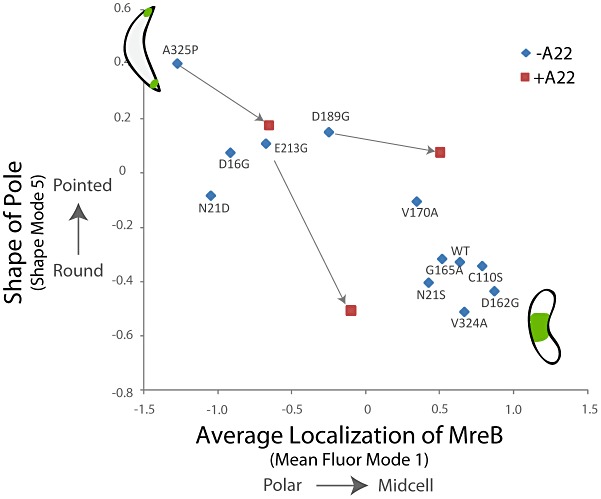
Recruitment of MreB to the cell poles correlates with pointed cell poles. For each strain, pole morphology (as measured with Shape mode 5) is plotted as a function of the subcellular distribution of MreB (average Fluor mode 1) in the absence of A22 (blue). For three of the mutants (A325P, E213G and D189G), both subcellular localization and pole shape change in the presence of A22 (red squares connected by arrows to the −A22 blue diamonds).

To determine to what extent the changes in localization of MreB could be correlated with changes in cell shape, we calculated the correlation coefficients for the mean value for each strain in each PCA shape mode and MreB localization. We found that the only metric of cell shape that is linearly related to the subcellular distribution of MreB is Shape mode 5, which corresponds to polar morphology (R = –0.87, *P* = 0.0002; Fig. 8; Fig. S16): cells with polar MreB have pointed poles. Average values in Shape modes 1–3 were found to have no significant linear correlation with the average subcellular distribution of MreB (*P* > 0.15, Fig. S16). Thus, strains with very similar MreB localization patterns do not necessarily have similar lengths, curvatures or average widths.

In the presence of A22, some of the strains – namely G165A, C110S, V324A and D16G – do not considerably change either their pole width or their subcellular localization of MreB in the presence of A22 (Fig. 6C and D and data not shown). These strains were also observed to have very similar cell shapes in the presence and absence of A22 (Figs S3, S5–9 and S11); again, we think it is likely that these mutant proteins have a low affinity for A22. In contrast, strains with A325P, E213G and D189G variants of MreB shift their localization of MreB towards midcell and away from the poles and develop rounder poles and more symmetric cell widths (Figs 6 and 8).

## Discussion

### A22-resistant *Caulobacter* strains have distinct cell shapes and MreB localization patterns

In this work, we quantitatively compared cell shape phenotypes and cell cycle regulated MreB localization patterns in a collection of *Caulobacter* strains bearing single point mutations in *mreB*. We determined that greater than 98% of the variation in cell shape is captured along five orthogonal principal component axes roughly corresponding to length, average width, curvature, cellular asymmetry and width of the poles. The average localization of MreB in these mutants was found to be either peaked at midcell, broadly distributed in the middle of the cell and away from the poles, uniformly distributed, or polar. The polar localization of MreB is strongly correlated with the development of pointed, as opposed to rounded, cell poles. Average cell length, width and curvature do not correlate with the average subcellular localization of MreB.

We propose that mutations that confer resistance to A22 also perturb other aspects of MreB function and dynamics. These changes could result from the mutation altering the kinetics of the nucleotide cycle (ATP binding, hydrolysis and release), the properties of self-assembly, or the binding of MreB to interacting proteins. Given that *mreB* is essential in *Caulobacter* and that all of the mutant strains grow with comparable doubling times and an approximately rod-like shape, it is reasonable to assume that none of these mutations completely destroy the ability of MreB to fold properly and perform its essential function in the cell. However, it is likely that the mutant proteins have subtle conformational changes in the structure that have consequences for protein activity and could provide novel insights into MreB's control of cell shape. For example, we found that defects in cell curvature and width do not always co-segregate: cells with similar cell curvatures do not necessarily have similar cell widths (Fig. 3). This result indicates that MreB has distinct and separable functions in at least two pathways for cell shape determination (width and curvature). We think it is likely that some of the mutations perturb interactions with distinct MreB binding partners, such as crescentin (Charbon *et al*., 2009) or RodZ (van den Ent *et al*., 2010). Thus, these mutant strains will be valuable tools for future work on the mechanism of MreB in these pathways. In addition, we identified three mutations that remain responsive to A22: D189G, E213G and A325P. Specifically, the presence of A22 partially rescues both the subcellular localization and cell shape (with respect to the shape of the poles) in these mutants. Thus, A22 may be used to further alter the dynamics of MreB polymerization in these strains, analogous to Benomyl-dependent β-tubulin mutations in yeast.

### MreB may also function at the poles in a wild-type cell

We identified four amino acid substitutions that cause MreB to localize to the poles. We propose that this phenotype points to a function for MreB at the cell poles even in wild-type cells. The poles of the *Caulobacter* cell are specialized subcellular regions that recruit specific molecular components in a cell cycle-dependent manner, including histidine kinases, the chemosensory apparatus, the machinery to build polar appendages (flagellum and stalk), and protein components that are required to anchor and segregate the chromosome (Goley *et al*., 2007). There is already evidence to suggest that MreB functions at the poles in *Caulobacter*. In wild-type *Caulobacter*, MreB partially and transiently localizes to the poles, especially early in the cell cycle after synchronization (Fig. 7 and unpublished observations by N.A.D.). In addition, it has been shown that MreB can co-immunoprecipitate with specific sequences of DNA near the origin of replication (Gitai *et al*., 2005), which is localized to the poles in *Caulobacter* and is anchored to the cell surface by PopZ and the partitioning protein ParB (Bowman *et al*., 2008; Ebersbach *et al*., 2008). Therefore, at least a small population of MreB in wild-type cells exists at the pole in close proximity with this region of the chromosome and the segregation machinery. MreB has also been implicated genetically in stalk biogenesis and growth (Gitai *et al*., 2004; Wagner *et al*., 2005), so it is possible that it is required at early points in the cell cycle to recruit factors that synthesize new cell wall at the stalk. Lastly, it has been observed that newborn *Caulobacter* cells have asymmetrically shaped poles, with the newest pole being more blunt and the oldest pole (formed from the division prior) being more pointed (Aaron *et al*., 2007). The transition from blunt to pointed is thought to occur gradually over the course of the cell cycle (Aaron *et al*., 2007). It is possible that MreB is involved in this process in wild-type cells, and that those mutants that are trapped at the poles continue to remodel the poles throughout the cell cycle to a greater extent than occurs in wild-type cells (which relocalize MreB to midcell). As a result, MreB and peptidoglycan synthetic enzymes would be depleted from midcell, potentially leading to variable cell widths and more pointed poles.

In these polar MreB mutants, it seems possible that cell growth is redirected from the lateral side walls to the poles. The Gram-positive species *Corynebacterium glutamicum* grows by adding new cell wall material to the polar regions, while keeping the lateral side walls inert (Daniel and Errington, 2003). In contrast, the Gram-negative *E. coli* and the Gram-positive *Bacillus subtilis* are thought to add new material along the sides and at the division plane, keeping the poles of the cell inert (de Pedro *et al*., 1997; Daniel and Errington, 2003; Varma *et al*., 2007). In *Caulobacter*, newly synthesized cell wall is found mainly at midcell, but a small amount of new material is inserted at the pole at the base of the stalk (Aaron *et al*., 2007). While these different species are known to exhibit distinct and characteristic growth patterns, no genetic manipulation has yet been shown to be capable of switching the mode of growth in a single organism. Future work will be aimed at determining precisely how the mutations identified here alter the normal pattern of cell wall growth in *Caulobacter*, potentially providing insight into the more general question of how peptidoglycan synthesis is spatially and temporally regulated.

### The role of nucleotide hydrolysis in regulating MreB function

Many members of the actin superfamily, even those that do not form filamentous homopolymers, are regulated by ATP-dependent conformational changes (Hurley, 1996). Accordingly, it seems likely that the nucleotide binding and hydrolysis cycle affects the conformational state, and ultimately the function, of MreB. In this work, we isolated several viable amino acid substitutions in conserved motifs of the actin fold that are important for nucleotide binding and hydrolysis, including two absolutely conserved aspartic acids (D16 and D162) and E140, which is required for hydrolysis in ParM and Hsc70 (Wilbanks *et al*., 1994; Garner *et al*., 2004). We also isolated mutations in key residues outside of these motifs that are in close proximity to the nucleotide in the crystal structure of *T. maritima* MreB (van den Ent *et al*., 2001): D189, D192, E213 and K216.

In yeast, the mutation of actin's D11 to alanine (corresponding to D16 in *Caulobacter*) or the double mutation of D154 and D157 (corresponding to D162 and G165) is lethal (Wertman *et al*., 1992). Likewise, the mutation of Q137 (corresponding to E140 in *Caulobacter*) in yeast actin does not support normal growth in the absence of wild-type protein (Belmont *et al*., 1999). In contrast, we found that in *Caulobacter*, numerous individual substitutions in the nucleotide binding pocket are capable of supporting life with a normal doubling time. While it remains to be proven biochemically that these mutant forms of MreB are not able to hydrolyse ATP at a normal rate, we think it is likely that the mechanism of hydrolysis by *Caulobacter* MreB resembles that of other members of the actin superfamily. Thus, it seems probable that a normal rate of ATP hydrolysis is not required for the essential function of MreB. D16G, D162G, D189G and E213G variants of MreB fail to localize robustly to midcell prior to division, however, indicating that ATP hydrolysis is likely to be required for the cell cycle-regulated relocalization of MreB from the cell sides to the division plane in wild-type *Caulobacter*.

**Figure 9:**
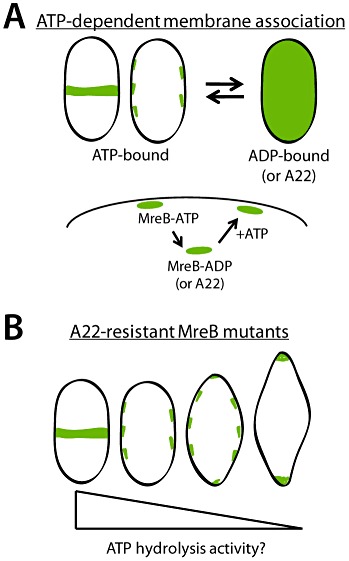
Model for the nucleotide dependence of MreB localization. A. We propose that ATP-bound MreB associates with the cell membrane in complexes (puncta and bands), whereas ADP-bound or A22-bound MreB is soluble in the cytoplasm. MreB localization is represented in green, in whole cells (top) or as an imaginary cross-section (bottom). B. We propose that the observed continuum of localization patterns for mutant MreB proteins represents a gradient of ATP hydrolysis activity, with the most inactive forms being those that localize to the poles.

Because there is evidence to suggest that A22 induces an ADP-like state *in vitro* in MreB of *T. maritima* (Bean *et al*., 2009) and causes the dissociation of the puncta and bands of wild-type MreB on the membrane of *Caulobacter* cells *in vivo* (Gitai *et al*., 2005), we propose that ATP-bound MreB is associated with the membrane and that ATP hydrolysis triggers the release of the protein back into the cytoplasm (Fig. 9A). Alterations in the nucleotide hydrolysis activity of MreB would alter its dynamic relocalization and steady-state cellular distribution but not necessarily its communication with the machinery that synthesizes the peptidoglycan cell wall. The association of MreB with the membrane is not expected to be direct, as MreB does not contain any obvious domains that could associate with the membrane. The membrane protein RodZ has been shown to bind directly to MreB and to be required for proper localization of MreB, but its interaction *in vitro* is not nucleotide-dependent (Alyahya *et al*., 2009; Bendezu *et al*., 2009; van den Ent *et al*., 2010). We think it is likely that additional unknown factors mediate the proposed nucleotide-dependent association of MreB with the membrane.

Because our mutants were isolated by selecting for resistance to a chemical that destabilizes MreB, we hypothesize that they all represent forms of the protein that more robustly favour self-association and assembly into patches on the membrane. Indeed, it has been shown previously in *B. subtilis* that a mutation at the position corresponding to *Caulobacter* D162 slows the dynamic reorganization of MreB (as assayed by photobleaching) (Defeu Soufo and Graumann, 2006). In our work, we observed a continuum of average subcellular localizations for the isolated MreB mutants, from peaked near midcell, to broadly peaked, to flat, to peaked at the poles (Fig. 6). We propose that this continuum of localization reflects a gradient of ATP hydrolysis activity, with the mutants that localize to the poles having the slowest rate of hydrolysis or an inability to change conformation in response to ATP hydrolysis (Fig. 9B). In at least two of the mutants, A325P and E213G, A22 is capable of releasing MreB from the pole and allowing it to become more evenly distributed (Fig. 6), suggesting that A22 is still able to bind these MreB mutants and act to partially restore the wild-type dynamics.

In support of this idea, some of the amino acids that were implicated in the variable width phenotype (and polar localization) appear to mediate subdomain contacts or would be predicted to perturb the local secondary structure (Fig. 5C). For example, E213 (E204 in *T. maritima*) on subdomain II forms a salt bridge with K58 (K49 in *T. maritima*) on subdomain I on the other side of the nucleotide cleft (van den Ent *et al*., 2001). Likewise, R185 appears to form a salt bridge with E119 to connect the two major domains of the protein (Fig. 5C). The mutation of A325 to a proline is likely to disrupt helix 12 and cause more severe consequences than the mutation to a threonine, which could potentially explain why A325P has a severe variable width phenotype, whereas A325T is relatively normal. Thus, it seems possible that the mutation of these particular residues has profound consequences for the conformational flexibility of the protein structure and the ability to be regulated by the nucleotide binding/hydrolysis cycle.

Our model makes predictions for the inherent biochemical parameters of self-assembly and nucleotide hydrolysis of the mutant MreB proteins *in vitro*, which should be testable using the biochemically tractable homologue of MreB from *T. maritima*. In addition, our model predicts that the fine-scale dynamic behaviour of the pattern of MreB localization in the cell is altered on relatively short timescales in these mutants (shorter than the entire cell cycle). This prediction can be tested by imaging single molecules of MreB or patches of MreB at intervals of a few seconds to minutes and using photobleaching techniques to assess the turnover of these patterns. Because mutations that are predicted to considerably slow ATP hydrolysis do not block cell growth but alter the subcellular localization of MreB and cell shape, we propose that the energy input (from ATP hydrolysis) is not required for MreB's contribution to cell wall synthesis or its assembly into clusters on the membrane but only for the dynamic large-scale patterning of cell wall growth.

## Experimental procedures

### Bacterial growth conditions

In this work, we grew *Caulobacter* only in rich media (PYE) at 28–30°C. Optical density was measured at 660 nm. A22 was synthesized according to (Gitai *et al*., 2005) and always used at a final concentration of 2.5 µg ml^−1^ on plates, in liquid, or in agarose pads. In our experience, the effect of A22 declines considerably with the age of the stock solution. Thus, we prepared fresh stock solutions before each experiment (never longer than two days prior). This practice helped ensure day-to-day reproducibility. In addition, we added A22 to liquid media only immediately prior to inoculation.

To synchronize the cells, 10 ml cultures in mid-log phase were spun at 10 000 *g* for 2 min in six microcentrifuge tubes. Cells were pooled into one tube and washed with ice cold M2 salts. Washed cells were resuspended in M2 salts and mixed with an equal volume of cold Percoll. Cells were then spun at 10 000 *g* for 20 min at 4°C. Stalk and predivisional cells were removed, and the bottom band of swarmer cells was isolated. Swarmers were washed twice with M2 salts and then resuspended in PYE and added immediately to microscope slides for imaging.

All of the strains used in this work are listed in Table S3.

### Selection of A22-resistant mutants

To select for A22 resistance, wild-type CB15N *Caulobacter* was grown overnight in PYE at 30°C to an OD at 660 nm ∼ 1.0. Solid PYE plates containing 2.5 µg ml^−1^ A22 were prepared by adding the appropriate amount of stock solution (in DMSO) to the molten media agar solution prior to pouring and solidifying. Overnight cultures were plated (200 µl) on A22-containing PYE plates and grown at 30°C for three days. At this point, ∼ 1–50 colonies (of varying sizes) were visible on each plate. One colony per plate was restreaked onto a fresh plate containing A22 and grown for another three days. Each strain was grown overnight in a 2 ml deep 96-well plate (USA Scientific) in 2 ml PYE containing A22. Cells were then frozen in 10% DMSO and stored at −80°C. The mutant Q26P was not isolated in our selection; it was obtained from the lab of Christine Jacobs-Wagner at Yale University (Aaron *et al*., 2007).

To identify mutations in *mreB*, we PCR-amplified the gene (with flanking regions on both sides) using a small amount of liquid overnight culture and primers ND232F and ND233R (Table S4). Products were purified with a 96-well PCR cleanup kit (Zymo Research) and sequenced by Sequetech (Santa Clara, CA, USA). The results of the sequencing are presented in Table S1. Strains that possessed more than one mutation in *mreB* were discarded. Additional strains were discarded because in subsequent experiments they were found to grow very irregularly, meaning that they exhibited high day-to-day variation in doubling time and morphology and often they exhibited highly variable colony size on a streak plate. We considered these strains unstable (susceptible to suppressor mutations). While these mutations may be interesting to study in the future, we chose to first consider only those strains that exhibit a stable growth pattern.

### Imaging the shapes of A22-resistant *Caulobacter*

Many of the mutant strains we isolated become very filamentous and pleiomorphic at high density (unpublished observations by N.A.D.). In order to get an accurate measure of the cell shapes of these strains in *steady-state* log phase (rather than the shapes they adapt as they are transitioning between different phases), we tried to prevent the strains from ever becoming too dense. All of the selected strains were grown from a frozen stock or colony inoculation in PYE at 30°C with or without A22 in a 2 ml 96-well plate (USA Scientific) to an OD of ∼ 0.3–0.4. Then, these cultures were diluted 10- to 20-fold into pre-warmed PYE media (with or without A22) in a Costar flat-bottom 96-well non-treated culture plate (Corning) covered with sterile microporous sealing film (USA Scientific). Growth was resumed on a shaker plate at 28°C. Density was monitored every 20–30 min with a 96-well plate reader spectrophotometer. Doubling time was calculated using density readings taken over a 2 h period (beginning ∼ 1 hr after dilution into the Costar plate). Half of the plate was diluted at a delay of 1–1.5 h, to account for the time it would take to image the first set of cells. For wild-type, which will not grow overnight in A22, we diluted the overnight culture grown in the absence of A22 into media containing A22 (grown for ∼ 4–6 h).

To image the cells we used medium-throughput techniques and materials that have been previously described (Christen *et al*., 2010), including a custom-built apparatus for making 48 agarose pads on a glass slide spaced at intervals compatible with a 96-well plate. Cells were imaged on pads of 1% agarose in M2G or water. A custom-built 48-well metal stamp was sterilized and then used to transfer half of the cells from the 96-well plate to these 48 agarose pads. A large piece of cover glass was placed on top, and the slide was sealed with thermoplastic (‘hot’) glue.

### Construction and growth of fluorescent strains

Fluorescent Venus fusions to MreB mutants were constructed by cloning the *mreB* gene into pXVENN-2 (Thanbichler *et al*., 2007). The gene was amplified from liquid overnight cultures of the mutant strain using primers to introduce NheI and BglII restriction sites (ND238F and ND239R, Table S4). These enzymes were used to move *mreB* into pXVENN-2, and the resulting plasmid (encodes resistance to Kanamycin) was electroporated into CB15N *Caulobacter*. This plasmid integrates into the genome at the xylose-regulated promoter (Thanbichler *et al*., 2007). The resulting merodiploid strains (JAT 860–870), containing a mixture of wild-type MreB (endogenous, unlabelled) and mutant (Venus-labelled under *Pxyl*), were used to assess the dominance of the Venus-mutant fusions (Fig. S13).

Phage transduction (CR30) was used to move the fluorescent construct from the wild-type background to the A22-resistant *mreB* background, selecting for Kanamycin resistance. Thus, the strains shown in Figs 6 and 7 (JAT 783–798) each have two copies of *mreB* encoded. One is unlabelled at the native locus, while the other encodes an N-terminal fusion to Venus at the xylose-inducible locus. Both copies carry identical mutations, and no wild-type protein is present. To image the fluorescent strains, they were grown overnight in PYE + Kan + 0.2% glucose with or without A22. Cells were then washed in equivalent media lacking glucose and grown for another ∼ 2 hours. At an OD of ∼ 0.1–0.2, xylose was added to 0.03%. Cells were allowed to grow for 1–1.5 hours in induction conditions and then imaged on 1% agarose/PYE with or without A22.

GFP–crescentin expressed from its endogenous promoter (as well as unlabelled crescentin) was transduced into a subset of the mutant strains, selecting for gentamicin resistance (construct characterized in Ausmees *et al*., 2003).

### Assessment of dominance of Venus-A22-resistant MreB variants over wild-type MreB

Strains JAT 860–870, encoding wild-type *mreB* under the native promoter and mutant MreB fused to Venus under the inducible *Pxyl*, were grown from a single colony to saturation overnight in PYE. Serial dilutions were made from 10^−1^ to 10^−6^ into fresh PYE media, and 5 µl of each dilution was spotted onto four PYE plates containing the following: 0.2% glucose, 0.2% glucose + A22, 0.2% xylose or 0.2% xylose + A22. After the spots dried, the plates were incubated at 30°C for 2 days and then photographed with backlit illumination. The amounts of growth observed on the plates containing 0.2% xylose or 0.2% glucose without A22 were indistinguishable (data not shown).

### Microscopy

To image the cell shapes of the unlabelled A22-resistant strains, we used a DM6000B automated microscope equipped with a 100 × 1.46 NA HCX Plan APO oil immersion objective (Leica) and a C9100 EM cooled CCD camera (Hamamatsu). Automated image acquisition was performed by KAMS-acquire software (Christen *et al*., 2010). The 48-well microscope slide was scanned across 8 × 6 rows, imaging four fields in each well before moving to the next. Manual refocusing was performed as needed.

To image the fluorescent strains, we used an upright fluorescence microscope (Zeiss, Thornwood, NY, USA) equipped with a plan-apo 100× phase 3 objective lens, conventional epi-fluorescence filter set and a 1024 × 1024 pixel back illuminated EMCCD camera (Andor, South Windsor, CT, USA).

For the figures, the brightness and contrast were adjusted and overlay images were produced with Adobe Photoshop.

### Analysis of cell shape in MreB mutant *Caulobacter*

Images of bacteria were segmented in Matlab (v. 2008a) with binary thresholding, followed by a custom marker-based watershed algorithm. For this algorithm, we chose parameters that seemed reasonable by eye to separate touching cells and recently divided cells. The parameters were kept constant for all images taken under the same set of conditions.

For the analysis of cell shape, we used Celltool, an open access software tool for measuring and analysing cell shape (Pincus and Theriot, 2007). Contours were extracted from the binary, segmented images of bacteria and resampled so that each cell was represented by a contour of 100 evenly spaced points. The shape modes depicted in Fig. 1D are the primary modes of variation in the data set of all cells of all strains (numbers and strains listed in Table S2). The distribution of values in each mode for each strain grown in the absence and presence of A22 are presented in Figs S5–9 as stair plots of normalized histograms, generated in Matlab. For each strain, histograms were normalized to the total number of cells analysed in each condition (+/−A22). Values in the range of −2 to 8 for Shape mode 1, −5 to 5 for Shape modes 2, 3 and 5, or 0 to 5 for Shape mode 4 were counted in 100 equally sized bins.

For Fig. S12, we calculated the mean value in each parameter for each strain and plotted every pairwise combination of shape metrics. Pearson's correlation coefficients and *P*-values for each pairwise combination were calculated using the CORR function in Matlab. Contour centerlines were determined as in Sycuro *et al*. (2010), allowing direct measurement of cell length (pole-to-pole distance along the centerline), width (distance between the right and left side of the contour, orthogonal to the centerline, averaged from the 10% to 90% position to exclude pole regions, noted as ‘width in middle’ in the figure), and polar width (as previous, but only in the pole regions, noted as ‘width at poles’ in the figure). Width ratio corresponds to (width in middle)/(width at poles). Cell curvature was measured directly by averaging the length-corrected geometric curvature of the contour in non-polar regions (Sycuro *et al*., 2010). Finally, asymmetry was measured by calculating the root mean squared deviation between contour points and the corresponding points of a vertically reflected copy of the same contour.

Note that the *ad hoc* geometric curvature metric cannot distinguish between S-type and C-type curvature. Indeed, an S-shaped cell could have a higher value of ‘curvature’ than a C-shaped cell (not shown). For *Caulobacter*, wild-type cells are C-shaped, whereas S-shaped cells are distinctly not wild-type and are likely to have defects in crescentin localization. Thus, the analysis of curvature in our mutant *Caulobacter* population is complicated, and the *ad hoc* geometric curvature metric is not well suited for this purpose. The PCA, however, nicely distinguishes between these two types of curvature: the transition between C-shaped and straight cells is represented by Shape mode 2, whereas the difference between S-shaped and C-shaped cells is measured by Shape mode 4. For this reason, we preferred the PCA Shape modes to the *ad hoc* metrics of cell shape for our analysis, particularly for analysing defects in curvature. Because Shape mode 4 correlates with Shape mode 1, we mostly used Shape mode 2 to measure ‘curvature’ in our analysis.

### Clustering MreB mutant strains by cell shape

The distribution of cell shapes from all conditions was summarized as a point distribution in five dimensions by PCA (Pincus and Theriot, 2007). The distribution was rescaled to have unit variances along each axis, though the results are not largely dependent on rescaling (not shown). To compare strains and treatment conditions, as opposed to individual cells, however, it is necessary to define a distance metric between sets of points in that five-dimensional space. To this end, we employed the ‘Earth Mover's Distance’ (Rubner *et al*., 2000), which poses the distance between two point-sets as a transportation problem: given the spatial locations of a set of ‘producers’ and a set of ‘consumers’ and the amount of production or consumption at each position, find a ‘transport plan’ to get the product to the consumer with as little spatial movement as possible. Such problems can be solved in closed form with the ‘transportation simplex method’ (Koopmans and Reiter, 1951). In the case that there are an equal number of cells in each treatment condition, the problem reduces to how to superimpose one set of cells on the other with as little movement (in the 5D cell-shape space) as possible; when there are unequal numbers of cells, the total amount of ‘production’ and ‘consumption’ is set to unity and spread equally among all of the cell positions, which will result in an assignment that optimally transforms one set of points on to the other, allowing for splitting and merging to account for the unequal numbers.

The total amount of transportation necessary to affect this transformation is then used as the ‘distance’ between any two point-sets (i.e. sets of cells). Pairwise distances were computed between all strains in the +A22 and −A22 treatment conditions separately. After computing these distances, average-linkage hierarchical clustering was applied to the distance matrix in order to visualize the relationships between the strains with and without A22. To set a threshold for defining reasonable clusters, we used the EMD to calculate the distance between the distribution of cells in each strain captured on day 1 to the distribution of cells from that same strain captured on day 2. This distance corresponds to the day-to-day variation. We defined a threshold for the cluster diagrams as the average amount of day-to-day variation for all samples.

### Analysis of A22's effect on cell shape in MreB mutants

The total variance in each strain in the presence and absence of A22 (Fig. S10) was measured by calculating the variance in X and Y positions in (µm^2^) for each point along the cell outlines across all bacteria measured, and summing those variances. (Equivalently, summing the traces of covariance matrices calculated for the X positions and Y positions of each of the points along the outline of the cells.)

To calculate the effect of A22 on cell shape in each strain, we used the EMD (described above) to calculate the distance between the distribution of cell shapes in the presence of A22 from the distribution of cells in that same strain grown in the absence of A22, precisely as the distances between different strains were calculated for hierarchical clustering (as above). This distance was calculated for each strain separately, and the results are shown in purple in Fig. S11. To make Fig. 2C, we set an arbitrary threshold for the extent of change in A22 that would separate ∼ 25% of the mutant residues that change the least in the presence of A22 from the rest of the strains. Relaxing this threshold includes residues that are more and more distant from the proposed A22-binding site.

To compare each strain to the wild-type (untreated) cell shape, we calculated the distance between each strain's distribution of cell shapes to the distribution of shapes in the wild-type (grown in the absence of A22). This distance was separately calculated for each strain grown in the presence and absence of A22 (in each case comparing the mutant to the wild-type untreated distribution). The difference between the distance from the mutant strain grown without A22 to wild-type and the distance from the mutant strain grown with A22 to wild-type is plotted in green in Fig. S11.

### Selection of strains to demonstrate the separation in curvature and width phenotypes

The strains highlighted in Fig. 3 were chosen to represent a range of curvature and width values. We selected pairs of strains at three different Shape mode 2 values. The three pairs represent curved, intermediate, and straight phenotypes. Each strain within a pair has similar Shape mode 2 values but different Shape mode 3 values. The average values of Shape mode 2 in these strains are as follows: G329C, 0.56; I163F, 0.54; E119G, −0.25; N21Y, −0.31; I266S, −0.58; D189G, −0.61. The average values of Shape mode 3 in these strains are as follows: G329C, 1.00; I163F, −0.49; E119G, 0.55; N21Y, −0.54; I266S, 0.06; D189G, −0.51. Similar results were obtained using the *ad hoc* metrics of curvature and cell width instead of the Shape modes (data not shown).

### Analysis of fluorescent crescentin and MreB distributions

The analysis of average crescentin and MreB distributions was performed with Celltool. As described above for the analysis of cell shape, cells were segmented in Matlab using the phase contrast images and contours were resampled to 100 points in Celltool. Then, we defined a 50-point centerline connecting the two most distant points in the contour, and matched each contour to its corresponding fluorescence image. This centerline defines the long axis of the cell. For MreB, we measured the intensity of fluorescence at each point on this centerline, averaging over the whole width of the cell. For crescentin, we did the opposite, averaging the values along the whole length of the cell at different points along the short axis of the cell. Specifically, the intensity of MreB or crescentin was measured with the axis-swath option of the measure_contours command, using the measurement mode length-profile or depth-profile respectively. The total intensity values were normalized to average intensity, so that the only difference between the profiles is the distribution of this intensity along the centerline. Given that the fluorescent reporter is under the same inducible promoter in all of these strains, we feel that this is a reasonable assumption. Furthermore, the shape of the one-dimensional strain profiles does not depend on fluorescence intensity (data not shown).

For each MreB-labelled strain, the following number of cells were averaged in −A22 and +A22 respectively: WT, 627 and 656; D16G, 539 and 518; N21S, 502 and 452; N21D, 556 and 425; C110S, 558 and 501; D162G, 556 and 561; G165A, 583 and 691; V170A, 506 and 571; D189G, 600 and 701; E213G, 523 and 432; V324A, 541 and 471; A325P, 674 and 571. For each crescentin-labelled strain, the following number of cells were averaged: WT, 1010; C110S, 734; D16G, 491; D162G, 457; D189G, 454; E119G, 369; V324A, 676. Randomly reducing the size of the data set to 300 cells per strain does not change the shapes of the profiles.

The PCA Fluor modes, the modes of variation in the fluorescent MreB profiles, were generated in Matlab. We first generated a matrix of all normalized MreB profiles (all cells of all strains) and then calculated the mean profile by averaging all values at each point. This mean was then subtracted from each profile, and the covariances of the resulting values (the deviations from the mean) were measured. The PCA Fluor modes are the eigenvectors of this covariance matrix. The values for each mode were normalized by subtracting the mean and dividing by the standard deviation of all values in that mode (to generate a Z-score). Figure S14A contains illustrations of profiles that represent the mean and one or two standard deviations from the mean in each axis. PCA Fluor mode 1 accounts for 35% of the total variation in all of the cell profiles and appears to capture the difference between localization at the poles and localization at midcell. The second primary mode appears to reflect asymmetry in the distribution of MreB from one end of the cell to the other and captures 16% of the total variation. The remaining PCA modes of fluorescence variation capture finer details in the profiles. Importantly, if we generate principal component axes using only the mean profiles for each strain, we obtain similar modes of variation (data not shown). In this case, however, almost 90% of the total variation can be explained by the first mode, which is identical to the Fluor mode 1 generated from all cells (data not shown), indicating that this mode is capable of distinguishing important variation between strains in the subcellular distribution of MreB. Representative cells from each localization class, their corresponding profiles, and their PCA mode values are shown in Fig. S14B to illustrate how these PCA describe the MreB patterns.

### Analysis of Venus–MreB time-lapses

Images were segmented as described above. While our segmentation algorithm was found to be far better than thresholding alone for separating newly divided cells, it is still difficult to accurately determine the exact point of division with phase contrast microscopy. Because we used similar parameters for the image acquisition and segmentation of all time-lapses, we believe that at least this error will be systematic and not specific to certain strains. Cells that were obviously not segmented correctly were not included in the analysis.

In Fig. 7, we report the intensity of MreB along the centerline at each time point as a Z-score. To calculate this value for each cell, at each point on the centerline we calculated the mean and standard deviation intensity across all time points. For each time point, we then subtracted the mean and divided by the standard deviation at each point to get a Z-score. To report this intensity as a function of cell length, rather than relative coordinate, we determined the length of each centerline segment in each cell (Fig. S15). Because each centerline consists of 50 segments, the length per segment is equal to the length of the cell divided by 50. Normalized PCA Fluor mode 1 values were calculated using the axes defined by all cells (described above). Randomly reducing the size of the data set from each strain to 44 cells (the lowest N used) does not affect the results presented in Fig. 7.

### Highlighting residues on the crystal structure

UCSF–Chimera was used to align the protein sequences of *T. maritima* and *Caulobacter* MreB and manipulate the crystal structure of *T. maritima* MreB1 complexed with AMPPNP (PDB: 1JCG; van den Ent *et al*., 2001). Note that some of the residues that we identified in *Caulobacter* are not conserved in *T. maritima* (for example, C110, Q26 and S181). These residues were changed in the *T. maritima* structure to match the *Caulobacter* sequence using the SWAPAA function before generating the images in Figs 2C and 4C.

For the alignment of MreB (*Caulobacter* and *T. maritima*) with yeast actin and bovine Hsc70 presented in Fig. S1, we used STRAP (Gille and Frommel, 2001; Gille *et al*., 2003).
