# Supplementary material for: Mutations in the nucleotide binding pocket of MreB can alter cell curvature and polar morphology in Caulobacter
Source: Mol Microbiol. 2011 May 26;81(2):368–94. doi: 10.1111/j.1365-2958.2011.07698.x (PMC3137890; doi:10.1111/j.1365-2958.2011.07698.x)

# Supplemental figure legends

**S1: Alignment of *Caulobacter* MreB with *T.maritima* MreB, yeast actin and bovine Hsc70.** Known structures for *T.maritima* MreB (PDB: 1JCE) (van den Ent et al., 2001), *S. cerevisiae* actin bound to gelsolin (1YAG) (Vorobiev et al., 2003) and bovine heat shock cognate protein 70 kD (Hsc70, 1HPM) (O'Brien & McKay, 1995) were aligned with the primary sequence of *Caulobacter* MreB using STRAP (Gille & Frommel, 2001). Highlighted residues are identical (blue) or similar (magenta) in at least three of the sequences. Bars above the sequences represent β-sheets, and coils represent α-helices. Blue shaded boxes highlight the regions corresponding to the conserved sequence motifs identified in (Bork et al., 1992). The sequences below each box correspond to the consensus sequence for each motif, where bold residues are absolutely conserved (in Phosphate I, the presence of an E substitution for D is allowed). Plus signs mark the residues identified as being in close proximity to the nucleotide in the crystal structure of *T.maritima* MreB (van den Ent et al., 2001). Asterisks mark nucleotides that have been shown in other systems to contribute to hydrolysis (see text). In the motifs, h=hydrophobic (VLIFWY), f=partly hydrophobic (VLIFWYMCGATKHR), t=tiny (GSAT), s=small (GSATNDVCP), and p=tiny plus polar (GSATNDQEKHR) (Bork et al., 1992). The substitutions studied in this work are shown with arrows above the *Caulobacter* sequence.

**S2: Doubling time does not vary considerably across A22-resistant *mreB* mutant strains.** The doubling times for all strains grown in PYE at 28ºC with (red) or without (blue) A22 were measured simultaneously, as described in the Experimental Procedures. Plotted are the means and standard deviations for five replicate experiments performed on different days. Asterisks denote strains with particularly variable doubling times and cellular morphology (See Supplemental Figure 3).

**S3: A22-resistant *mreB* mutant *Caulobacter* strains have subtly distinct morphologies.** Shown are representative cells of 35 strains of *Caulobacter* bearing unique mutations in *mreB* grown in rich media in the absence (A) or presence (B) of 2.5 μg/ml A22. From left to right, top to bottom, strains are placed in order of the mutated amino acid, with wild type in the bottom right corner. Labels are color coded according to the morphological clustering presented in

Figure 2. Scale bars represent 5 µm.

**S4: Mutation replicates have very similar morphologies.** Shown are representative cells of two "mutation replicates" (see text) for each of the most common mutations. Strains were grown in the absence of A22. From left to right, top to bottom, strains are placed in order of the mutated amino acid. Images on left and right for each mutation correspond to "a" and "b" mutation replicates strains. Scale bar represents 5 µm.

**S5: Distributions of Shape mode 1 values.** Histogram traces of the values in Shape mode 1 (roughly corresponding to cell length) for each analyzed strain grown in the absence (blue) and presence (red) of A22. Values were binned into 100 equally sized bins from -2 to 8. Each histogram was normalized to the total number of cells in that sample (the Y-axis corresponds to the fraction of total N). Strains are shown in alphabetical order.

**S6: Distributions of Shape mode 2 values.** Histogram traces of the values in Shape mode 2 (corresponding to C-shaped curvature) for each analyzed strain grown in the absence (blue) and presence (red) of A22. Values were binned into 100 equally sized bins from -5 to 5. Each histogram was normalized to the total number of cells in that sample (the Y-axis corresponds to the fraction of total N). Strains are shown in alphabetical order.

**S7: Distributions of Shape mode 3 values.** Histogram traces of the values in Shape mode 3 (corresponding to cell width) for each analyzed strain grown in the absence (blue) and presence (red) of A22. Values were binned into 100 equally sized bins from -5 to 5. Each histogram was normalized to the total number of cells in that sample (the Y-axis corresponds to the fraction of total N). Strains are shown in alphabetical order.

**S8: Distributions of Shape mode 4 values.** Histogram traces of the absolute values in Shape mode 4 (corresponding to S-shaped curvature) for each analyzed strain grown in the absence (blue) and presence (red) of A22. Values were binned into 100 equally sized bins from 0 to 5. Each histogram was normalized to the total number of cells in that sample (the Y-axis corresponds to the fraction of total N). Strains are shown in alphabetical order.

**S9: Distributions of Shape mode 5 values.** Histogram traces of the values in Shape mode 5 (corresponding to pole shape or variation in overall cell width) for each analyzed strain grown in the absence (blue) and presence (red) of A22. Values were binned into 100 equally sized bins from -5 to 5. Each histogram was normalized to the total number of cells in that sample (the Y-axis corresponds to the fraction of total N). Strains are shown in alphabetical order.

**S10: Growth in A22 decreases the variance in cell shape.** Overall variance in cell shape across a population is plotted for each strain grown in the absence (blue) or presence (red) of A22. Black lines to the left of the sample name highlight "mutation replicates". Green lines highlight "culture replicates". From top to bottom, samples are in order of the mutated amino acid, with wild type at the top. Dotted blue line represents the amount of variation observed in wild type (WT1) grown in the absence of A22. In the absence of A22, all mutant strains exhibit larger variations in cell shape than wild type.

**S11: A22 affects cell shape more strongly in some *mreB* mutant strains.** To assess the response of the mutant strains to A22, we calculated the distance between the distribution of cells in each strain grown in the absence of A22 to the distribution of cells in that same strain grown in the presence of A22 (See Experimental Procedures). This distance for each strain is shown in purple. To assess whether or not A22 makes the cell shapes closer to or further from the wild type shape, we first calculated the distance between the distributions of cells in each strain grown without A22 to the distribution of cells in the untreated wild type population. The same comparison was made between cells grown in the presence of A22 and wild type cells grown in the absence of A22 (See Experimental Procedures). Because most cells were found to be closer to wild type in the presence of A22, we subtracted the second value from the first, and the result is shown in green. If the cells are more like wild type in A22, this number is positive. If they are less like wild type, this number is negative. Black lines to the left of the sample name highlight "mutation replicates". Orange lines highlight "culture replicates". From top to bottom, samples are in order of the mutated amino acid. Dotted gray line indicates the ~25% threshold used to make Figure 2C. This line separates the ~25% of amino acids that when mutated produce cells that change the least in the presence of A22.

**S12: Shape metrics are mostly uncorrelated in the *mreB* mutant collection.** Correlation matrix wherein each shape metric is plotted against every other shape metric. The metrics include both the PCA Shape modes (1-5) and the *ad hoc* measurements of shape (area, length, width at middle, width at poles, width ratio, and asymmetry; See Experimental Procedures). Each datapoint represents a given strain's average value in parameter (x) plotted against its average value in parameter (y). Red data points correspond to strains grown in A22, and blue data points correspond to strains grown in the absence of A22. The column labels correspond to the X-axes, and the row labels correspond to the Y-axes. To simplify visualization, the parameter values were normalized by subtracting by the mean and dividing by the standard deviation. For all plots, the axes range from -4 to +4. The Pearson correlation coefficient, calculated using all values (+/-A22), is presented for each pairwise comparison. The size of the font relates to the level of significance: the largest text corresponds to $p < 10^{-10}$, the intermediate size corresponds to $p <$ the Bonferroni-corrected threshold of 0.0002, and the smallest font denotes R-values that are not significant ($p > 0.0002$). A22-treated wild type was removed from this analysis, as it is a clear outlier.

**S13: Venus fusions to selected A22-resistant MreB mutants are functional and dominant to wild type.** Merodiploid strains (JAT783, 860-870) each encoding two copies of *mreB*, one endogenous wild type copy expressed from the endogenous promoter and another mutant copy fused to the fluorescent protein *venus* and expressed from the xylose-inducible promoter, were grown on glucose without A22 (A), glucose with A22 (B), or xylose with A22 (C). Growth of each strain under each condition is shown as a dilution series after two days of growth at 30°C. The xylose promoter, which is driving the expression of the only mutant copy of MreB in these strains, is induced with xylose and repressed with glucose. All strains except wild type are capable of growing on xylose with A22, indicating that the mutant Venus-fusions are functional and dominant to wild type with respect to growth on A22. Growth on xylose without A22 was indistinguishable from growth on glucose without A22 (data not shown).

**S14: PCA can be used to measure the variation in MreB localization between strains.** (A) First four principal modes of variation in the fluorescence intensity of MreB along the centerline.

The one-dimensional normalized intensity of MreB along a 50-point centerline was measured in every cell as described in the Experimental Procedures. Principal components analysis was performed on the profiles from every cell in all of the 12 labeled strains. Shown are profiles that represent the mean and one or two standard deviations from that mean in either direction of each principal axis. (B) Demonstration of how PCA Fluor Modes 1 and 2 capture the MreB intensity profiles in six example cells. (i). Images of six actual cells pulled from the dataset used to generate the PCA Fluor modes and their corresponding points in a graph of Fluor mode 1 vs. the absolute value of Fluor mode 2. Fluor mode 1 roughly describes the continuum from polar to peaked, whereas |Fluor mode 2| corresponds to the extent of asymmetry along the centerline. (ii) MreB intensity profiles for the images of cells shown in (i).

**S15: Timelapse data represented in absolute coordinates.** Intensity of MreB along the centerline over the course of the cell cycle for the individual cells presented in Figure 7 and Supplemental Movies 5-9. In these plots, MreB localization is presented in absolute coordinates (distance from one pole in micrometers) rather than in relative coordinates (distance along a 50-point centerline, as in Figure 7).

**S16: Subcellular distribution of MreB mutants correlates only with Shape mode 5.** For each analyzed strain, the average value in each Shape mode is plotted as a function of the subcellular distribution of MreB (average Fluor mode 1) in the absence (A) or presence of A22 (B). Pearson's correlation coefficients for each plot are presented in the top right corner. Only the relationships between Shape mode 5 and Fluor mode 1 (bold) are significant ($p$=0.0002 for -A22 and $p$=0.04 for +A22; $p$ >0.15 for all other comparisons).

## Supplemental movie legends

**SM 1:** Timelapse phase-contrast imaging of a single cell of the R185V186 duplication strain (JAT813 unsynchronized) on a PYE+1% agarose pad without A22. Images were acquired at 10 min intervals, and the movie is played at a rate of 6 frames/sec. This cell corresponds to that shown in Figure 4B(i). The scale bar in the first frame corresponds to 2 µm.

**SM 2:** Timelapse phase-contrast imaging of a single cell of the R185V186 duplication strain (JAT813, unsynchronized) on a PYE+1% agarose pad without A22. Images were acquired at 10 min intervals, and the movie is played at a rate of 6 frames/sec. This cell corresponds to that shown in Figure 4B(ii). The scale bar corresponds to 2 µm.

**SM 3:** Timelapse phase-contrast imaging of a single cell of the R185V186 duplication strain (JAT813, unsynchronized) on a PYE+1% agarose pad without A22. Images were acquired at 10 min intervals, and the movie is played at a rate of 6 frames/sec. This cell corresponds to that shown in Figure 4B(iii). The scale bar corresponds to 2 µm.

**SM 4:** Timelapse phase-contrast imaging of a single cell of wild type *Caulobacter* (CB15N, unsynchronized) on a PYE+1% agarose pad (no A22). Images were acquired at 10 min intervals, and the movie is played at a rate of 6 frames/sec. This cell corresponds to that shown in Figure 4B(iv). The scale bar corresponds to 2 µm.

**SM 5:** Timelapse imaging of Venus-MreB$^{WT}$ (JAT783) using phase-contrast and fluorescence microscopy. Images were acquired at 15 min intervals, and the movie is played at a rate of 4 frames/sec. Scale bar corresponds to 2 µm.

**SM 6:** Timelapse imaging of Venus-MreB$^{G165A}$ (JAT790) using phase-contrast and fluorescence microscopy. Images were acquired at 15 min intervals, and the movie is played at a rate of 4 frames/sec. Scale bar corresponds to 2 µm.

**SM 7:** Timelapse imaging of Venus-MreB$^{V324A}$ (JAT797) using phase-contrast and fluorescence microscopy. Images were acquired at 15 min intervals, and the movie is played at a rate of 4 frames/sec. Scale bar corresponds to 2 µm.

**SM 8:** Timelapse imaging of Venus-MreB$^{D189G}$ (JAT793) using phase-contrast and fluorescence microscopy. Images were acquired at 15 min intervals, and the movie is played at a rate of 4 frames/sec. Scale bar corresponds to 2 µm.

**SM 9:** Timelapse imaging of Venus-MreB$^{A325P}$ (JAT798) using phase-contrast and fluorescence microscopy. Images were acquired at 15 min intervals, and the movie is played at a rate of 4 frames/sec. Scale bar corresponds to 2 µm.

## References


Aaron, M., G. Charbon, H. Lam, H. Schwarz, W. Vollmer & C. Jacobs-Wagner, (2007) The tubulin homologue FtsZ contributes to cell elongation by guiding cell wall precursor synthesis in Caulobacter crescentus. *Mol Microbiol* **64**: 938-952.

Ausmees, N., J. R. Kuhn & C. Jacobs-Wagner, (2003) The bacterial cytoskeleton: an intermediate filament-like function in cell shape. *Cell* **115**: 705-713.

Bork, P., C. Sander & A. Valencia, (1992) An ATPase domain common to prokaryotic cell cycle proteins, sugar kinases, actin, and hsp70 heat shock proteins. *Proc Natl Acad Sci U S A* **89**: 7290-7294.

Gille, C. & C. Frommel, (2001) STRAP: editor for STRuctural Alignments of Proteins. *Bioinformatics* **17**: 377-378.

O'Brien, M. C. & D. B. McKay, (1995) How potassium affects the activity of the molecular chaperone Hsc70. I. Potassium is required for optimal ATPase activity. *J Biol Chem* **270**: 2247-2250.

van den Ent, F., L. A. Amos & J. Lowe, (2001) Prokaryotic origin of the actin cytoskeleton. *Nature* **413**: 39-44.

Vorobiev, S., B. Strokopytov, D. G. Drubin, C. Frieden, S. Ono, J. Condeelis, P. A. Rubenstein & S. C. Almo, (2003) The structure of nonvertebrate actin: implications for the ATP hydrolytic mechanism. *Proc Natl Acad Sci U S A* **100**: 5760-5765.

**Supplemental Table 1:** isolated A22-resistant mutations in *mreB* listed in order of sequence. Shading indicates strains that were discarded because they harbored more than one mutation or because they exhibited reduced or variable growth rates +/- A22.

| **Mutation in *mreB* ORF** | **Amino acid** | **# isolates** | **Conserved Motif** |
|---|---|---|---|
| C41T | A14V | 1 | Phosphate I |
| A47G | D16G | 6 | Phosphate I |
| C59T | A20V | 1 | Phosphate I |
| A61G | N21D | 2 | Phosphate I |
| A61T | N21Y | 1 | Phosphate I |
| A62G | N21S | 8 | Phosphate I |
| T68A | L23Q | 1 | Phosphate I |
| T68C | L23P | 1 | Phosphate I |
| T92A | I31N | 1 | Phosphate I |
| T158A | V53E | 1 | |
| T320A | V107E | 1 | |
| T328A | C110S | 7 | |
| A356G | E119G | 1 | |
| A367T | I123F | 1 | |
| A419C | E140A | 1 | Connect I |
| A419G | E140G | 1 | Connect I |
| C422T | P141L | 1 | Connect I |
| C431T | A144V | 1 | Connect I |
| A485G | D162G | 4 | Phosphate II |
| A487T | I163F | 1 | Phosphate II |
| G493A | G165S | 1 | Phosphate II |
| G494C | G165A | 1 | Phosphate II |
| A499G | T167A | 11 | Phosphate II |
| T509C | V170A | 1 | Phosphate II |
| C542T | S181L | 2 | Phosphate II |
| 555_GTCCGC | R185V186 duplication | 1 | |
| A566G | D189G | 3 | |
| A566T | D189V | 1 | |
| A575G | D192G | 9 | |
| A575T | D192V | 1 | |
| A638G | E213G | 1 | |
| A647G | K216R | 1 | |
| T797G | I266S | 1 | |
| C961T | L321F | 1 | Connect II |
| T971A | V324E | 1 | Connect II |
| T971C | V324A | 4 | Connect II |
| G973A | A325T | 1 | Connect II |
| G973C | A325P | 2 | Connect II |
| G985T | G329C | 1 | Connect II |
| T304A+T328A | F102I+ C110S | 1 | |
| T328A+T971C | V324A+ C110S | 1 | |
| T971C+T982C | V324A+ C328S | 1 | |

**Supplemental Table 2:** number of cells analyzed for determination of shape parameters. Strains are listed in order of sequence.

| | Day 1 | | Day 2 | | Total | |
|---|---|---|---|---|---|---|
| | **NoA22** | **A22** | **NoA22** | **A22** | **NoA22** | **A22** |
| **A14V** | 194 | 302 | 387 | 243 | 581 | 545 |
| **D16Ga** | 123 | 324 | 370 | 334 | 493 | 658 |
| **D16Ga2** | | | 456 | 431 | 456 | 431 |
| **D16Gb** | 305 | 312 | | | 305 | 312 |
| **D16Gc** | | | 424 | 395 | 424 | 395 |
| **N21D** | 136 | 293 | 412 | 275 | 548 | 568 |
| **N21Sa** | 169 | 250 | 342 | 335 | 511 | 585 |
| **N21Sb** | 349 | 350 | | | 349 | 350 |
| **N21Sc** | | | 435 | 354 | 435 | 354 |
| **N21Y** | 219 | 181 | 257 | 274 | 476 | 455 |
| **L23P** | 247 | 250 | 365 | 355 | 612 | 605 |
| **L23Q** | 300 | 283 | 306 | 218 | 606 | 501 |
| **Q26P** | 382 | 362 | 320 | 343 | 702 | 705 |
| **I31N** | 153 | 338 | 314 | 234 | 467 | 572 |
| **C110Sa** | 260 | 262 | 413 | 325 | 673 | 587 |
| **C110Sb** | 457 | 312 | 422 | 388 | 879 | 700 |
| **E119G** | 169 | 184 | 409 | 344 | 578 | 528 |
| **I123F** | 297 | 311 | 306 | 309 | 603 | 620 |
| **E140A** | 343 | 262 | 258 | 223 | 601 | 485 |
| **E140G** | 274 | 243 | 278 | 246 | 552 | 489 |
| **P141L** | 299 | 285 | 292 | 417 | 591 | 702 |
| **D162Gc** | 254 | 278 | 402 | 303 | 656 | 581 |
| **D162Ga** | 155 | 355 | 502 | 222 | 657 | 577 |
| **D162Gb** | 393 | 406 | | | 393 | 415 |
| **I163F** | 474 | 209 | 229 | 331 | 703 | 540 |
| **G165A** | 300 | 311 | 369 | 356 | 669 | 667 |
| **G165S** | 364 | 378 | 275 | 295 | 639 | 673 |
| **T167Aa** | 323 | 325 | 195 | 365 | 518 | 690 |
| **T167Ab** | 291 | 356 | 270 | 245 | 561 | 601 |
| **T167Ab2** | 193 | 282 | 327 | 266 | 520 | 548 |
| **V170A** | 301 | 447 | 262 | 204 | 497 | 651 |
| **S181La** | 333 | 233 | 335 | 351 | 668 | 584 |
| **S181Lb** | 351 | 285 | 306 | 221 | 657 | 506 |
| **D189Ga** | 246 | 274 | 374 | 231 | 620 | 505 |
| **D189Gb** | 289 | 263 | 396 | 228 | 685 | 491 |
| **D189V** | | | 334 | 366 | 334 | 366 |
| **D192Ga** | | | 452 | 434 | 452 | 434 |
| **D192Gb** | | | 286 | 490 | 286 | 490 |
| **D192V** | 296 | 322 | 283 | 404 | 579 | 726 |
| **E213G** | 323 | 364 | 263 | 330 | 586 | 694 |
| **K216R** | 269 | 273 | 278 | 409 | 547 | 682 |
| **I266S** | 261 | 324 | 316 | 384 | 577 | 708 |
| **V324Aa** | 317 | 272 | 212 | 248 | 529 | 520 |
| **V324Ab** | 273 | 463 | 297 | 315 | 571 | 778 |
| **V324E** | 218 | 214 | 324 | 336 | 542 | 550 |
| **A325Pa** | 298 | 416 | 367 | 368 | 668 | 784 |
| **A325Pb** | 360 | 282 | 452 | 325 | 812 | 607 |
| **A325T** | 307 | 290 | 373 | 420 | 680 | 710 |
| **G329S** | 331 | 216 | 335 | 258 | 666 | 474 |
| **WT** | 215 | 319 | 303 | 262 | 518 | 581 |
| **WT2** | 90 | 211 | 443 | 270 | 533 | 481 |
| **Sum** | **12501** | **13472** | **16326** | **15280** | **28765** | **28761** |

**Supplemental Table 3:** Strains used in this work.

| Name | Background | Relevant Genotype | Resistance | Reference |
|---|---|---|---|---|
| JAT661 | CB15N | *PmreB::A14V-mreB* | A22 | This work |
| JAT662 | CB15N | *PmreB::D16Ga-mreB* | A22 | This work |
| JAT663 | CB15N | *PmreB::D16Gc-mreB* | A22 | This work |
| JAT664 | CB15N | *PmreB::N21D-mreB* | A22 | This work |
| JAT665 | CB15N | *PmreB::N21Y-mreB* | A22 | This work |
| JAT666 | CB15N | *PmreB::N21Sa-mreB* | A22 | This work |
| JAT667 | CB15N | *PmreB::N21Sc-mreB* | A22 | This work |
| JAT668 | CB15N | *PmreB::L23Q-mreB* | A22 | This work |
| JAT669 | CB15N | *PmreB::L23P-mreB* | A22 | This work |
| JAT670 | CB15N | *PmreB::Q26P-mreB* | A22 | (Aaron et al., 2007) |
| JAT671 | CB15N | *PmreB::I31N-mreB* | A22 | This work |
| JAT672 | CB15N | *PmreB::C110Sa-mreB* | A22 | This work |
| JAT673 | CB15N | *PmreB::C110Sb-mreB* | A22 | This work |
| JAT674 | CB15N | *PmreB::E119G-mreB* | A22 | This work |
| JAT675 | CB15N | *PmreB::I123F-mreB* | A22 | This work |
| JAT676 | CB15N | *PmreB::E140A-mreB* | A22 | This work |
| JAT677 | CB15N | *PmreB::E140G-mreB* | A22 | This work |
| JAT678 | CB15N | *PmreB::P141L-mreB* | A22 | This work |
| JAT679 | CB15N | *PmreB::D162Gc-mreB* | A22 | This work |
| JAT680 | CB15N | *PmreB::D162Ga-mreB* | A22 | This work |
| JAT681 | CB15N | *PmreB::I163F-mreB* | A22 | This work |
| JAT682 | CB15N | *PmreB::G165S-mreB* | A22 | This work |
| JAT683 | CB15N | *PmreB::G165A-mreB* | A22 | This work |
| JAT684 | CB15N | *PmreB::T167Ab-mreB* | A22 | This work |
| JAT685 | CB15N | *PmreB::T167Aa-mreB* | A22 | This work |
| JAT686 | CB15N | *PmreB::V170A-mreB* | A22 | This work |
| JAT687 | CB15N | *PmreB::S181La-mreB* | A22 | This work |
| JAT688 | CB15N | *PmreB::S181Lb-mreB* | A22 | This work |
| JAT689 | CB15N | *PmreB::D189Ga-mreB* | A22 | This work |
| JAT690 | CB15N | *PmreB::D189Gb-mreB* | A22 | This work |
| JAT691 | CB15N | *PmreB::D189V-mreB* | A22 | This work |
| JAT692 | CB15N | *PmreB::D192Ga-mreB* | A22 | This work |
| JAT693 | CB15N | *PmreB::D192Gb-mreB* | A22 | This work |
| JAT694 | CB15N | *PmreB::D192V-mreB* | A22 | This work |
| JAT695 | CB15N | *PmreB::E213G-mreB* | A22 | This work |
| JAT696 | CB15N | *PmreB::K216R-mreB* | A22 | This work |
| JAT697 | CB15N | *PmreB::I266S-mreB* | A22 | This work |
| JAT698 | CB15N | *PmreB::V324E-mreB* | A22 | This work |

| | | | | |
|---|---|---|---|---|
| JAT699 | CB15N | *PmreB::V324Ab-mreB* | A22 | This work |
| JAT700 | CB15N | *PmreB::V324Aa-mreB* | A22 | This work |
| JAT701 | CB15N | *PmreB::A325T-mreB* | A22 | This work |
| JAT702 | CB15N | *PmreB::A325Pb-mreB* | A22 | This work |
| JAT703 | CB15N | *PmreB::A325Pa-mreB* | A22 | This work |
| JAT704 | CB15N | *PmreB::G329C-mreB* | A22 | This work |
| JAT783 | CB15N | *PmreB:mreB; Pxyl::Venus-mreB* | Km | This work |
| JAT784 | JAT662 | *PmreB:D16GmreB; Pxyl::Venus-D16GmreB* | A22+Km | This work |
| JAT785 | JAT666 | *PmreB:N21SmreB; Pxyl::Venus-N21SmreB* | A22+Km | This work |
| JAT786 | JAT664 | *PmreB:N21DmreB; Pxyl::Venus-N21DmreB* | A22+Km | This work |
| JAT787 | JAT672 | *PmreB:C110SmreB; Pxyl::Venus-C110SmreB* | A22+Km | This work |
| JAT789 | JAT679 | *PmreB:D162GmreB; Pxyl::Venus-D162GmreB* | A22+Km | This work |
| JAT790 | JAT683 | *PmreB:G165AmreB; Pxyl::Venus-G165AmreB* | A22+Km | This work |
| JAT792 | JAT686 | *PmreB:V170AmreB; Pxyl::Venus-V170AmreB* | A22+Km | This work |
| JAT793 | JAT689 | *PmreB:D189GmreB; Pxyl::Venus-D189GmreB* | A22+Km | This work |
| JAT795 | JAT695 | *PmreB:E213GmreB; Pxyl::Venus-E213GmreB* | A22+Km | This work |
| JAT797 | JAT699 | *PmreB:V324AmreB; Pxyl::Venus-V324AmreB* | A22+Km | This work |
| JAT798 | JAT702 | *PmreB:A325PmreB; Pxyl::Venus-A325PmreB* | A22+Km | This work |
| JAT813 | LS3814 | *PmreB:R185V186duplication-mreB; Pxyl:gfp-mreB* | A22+Km | This work |
| | CJW3805 | *PcreS:creS, creS-gfp* | Gm | (Ausmees et al., 2003) |
| JAT800 | JAT662 | *PmreB::D16G-mreB; PcreS:creS, creS-gfp* | A22+Gm | This work |
| JAT801 | JAT672 | *PmreB::C110S-mreB; PcreS:creS, creS-gfp* | A22+Gm | This work |
| JAT802 | JAT679 | *PmreB::D162G-mreB; PcreS:creS, creS-gfp* | A22+Gm | This work |
| JAT803 | JAT683 | *PmreB::G165A-mreB; PcreS:creS, creS-gfp* | A22+Gm | This work |
| JAT804 | JAT690 | *PmreB::D189G-mreB; PcreS:creS, creS-gfp* | A22+Gm | This work |
| JAT805 | JAT674 | *PmreB::E119G-mreB; PcreS:creS, creS-gfp* | A22+Gm | This work |
| JAT806 | JAT699 | *PmreB::V324Ab-mreB; PcreS:creS, creS-gfp* | A22+Gm | This work |
| JAT860 | CB15N | *PmreB::mreB; Pxyl::Venus-D16GmreB* | Km | This work |
| JAT861 | CB15N | *PmreB::mreB; Pxyl::Venus-N21SmreB* | Km | This work |
| JAT862 | CB15N | *PmreB::mreB; Pxyl::Venus-N21DmreB* | Km | This work |
| JAT863 | CB15N | *PmreB::mreB; Pxyl::Venus-C110SmreB* | Km | This work |
| JAT864 | CB15N | *PmreB::mreB; Pxyl::Venus-D162GmreB* | Km | This work |
| JAT865 | CB15N | *PmreB::mreB; Pxyl::Venus-G165AmreB* | Km | This work |
| JAT866 | CB15N | *PmreB::mreB; Pxyl::Venus-V170AmreB* | Km | This work |
| JAT867 | CB15N | *PmreB::mreB; Pxyl::Venus-D189GmreB* | Km | This work |
| JAT868 | CB15N | *PmreB::mreB; Pxyl::Venus-E213GmreB* | Km | This work |
| JAT869 | CB15N | *PmreB::mreB; Pxyl::Venus-V324AmreB* | Km | This work |
| JAT870 | CB15N | *PmreB::mreB; Pxyl::Venus-A325PmreB* | Km | This work |

**Supplemental Table 4:** PCR primers used in this work. Capitalized letters match the target sequence exactly; lowercase letters were added to the primer to facilitate cloning.

| Name | Sequence (5'-3') |
|---|---|
| ND232F | CAGAGATGGATTTGGCCCTTCTT |
| ND233R | GTCCACGTCAATGGCACCTTC |
| ND238F | tgacagatctTTCTCTTCCCTTTTCGGCGTGATCT |
| ND239R | tgacgctagcCTAGGCCAGCGTGGATTCCAGGAC |

## Supplemental Figure 1

```
                               V  G      S Q/P  P         N
                               14 16     21 23  26        31
MreB Caulobacter     1  MFSSLFGVISNDIAIDLGTANTLIYQKGKG...IVLN......EPSVVALRNVGG.....RKVVH
MreB Thermatoga      1  .....mlr..KDIGIDLGTANTLVFLRGKG...IVVN......EPSVIAIDSTT.......GEIL
Actin yeast          1  .....mdsEVAALVIDNGSGMCKAGFAGD..DAPRA......VFPSIVGRPRHQGIMVGMGQKDS
Hsc70 bovine         1  .....msKG.PAVGIDLGTTYSCVGVFQHGKVEIIANDQGNRTTPSYVAFTD..........TER
                                    fxfDxGssxxhhxhxpxp
                                       E
                                       *          Phosphate I

MreB Caulobacter    52  AVGIEAKQMLGRTPGHMEAIRPMRDGVIA....................................
MreB Thermatoga     43  KVGLEAKNMIGKTPATIKAIRPMRDGVIA....................................
Actin yeast         53  YVGDEAQSK...RG.ILTLRYPIEHGIVT....................................
Hsc70 bovine        50  LIGDAAKNQVAMN...PTNTVFDA..KRLIGRRFDDAVVQSDMKHWPFMVVNDAGRPKVQVEYKG

                                                     S           G    F
                                                     110         119  123
MreB Caulobacter    81  ......DFEVAEEMIKYFIRKVH....NRKGFVNPKVIVCVPSGATAVERRAINDSCL.NAGARR
MreB Thermatoga     72  ......DYTVALVMLRYFINKAK....GGMNLFKPRVVIGVPIGITDVERRAILDAGL.EAGASK
Actin yeast         78  ......NWDDMEKIWHHTFYNEL....RV.APEEHPVLLTEAPMNPKSNREKMTQIMFETFNVPA
Hsc70 bovine       110  ETKSFYPEEVSSMVLTKMKEIAEAYLGKT....VTNAVVTVPAYFNDSQRQATKDAGT.IAGLNV
                                                                                      hxx

                           G/A L                   G F  A  A   A               L    RVR  G/V
                            140                    162  165 167 170            181  185  189
MreB Caulobacter   135  VGLIDEPMAAAIGAGLPIHEP..TGSMVVDIGGGTTEVAVLSLS...GIVY...SRSVR.VGGDK
MreB Thermatoga    126  VFLIEEPMAAAIGSNLNVEE..PSGNMVVDIGGGTTEVAVISL....GSIV..TWESIR.IAGDE
Actin yeast        132  FYVSIQAVLSLYSSGRT.......TGIVLDSGDGVTHVVPIYA....GFSLPHAILRID.LAGRD
Hsc70 bovine       170  LRIINEPTAAAIAYGLDKKVGAERNVLIFDLGGGTFDVSILTIEDGIFEVK..STAGDTHLGGED
                        xxhhxptxxshfpxh             hffDxGtsthpxtxf                      +
                           *    Connect I              *      * Phosphate II

                        G/V                                          G  R
                        192                                         213 216
MreB Caulobacter   191  MDEATISYMRRHHN.............LLIG...ETTAERIKKEIGTARAP.........ADGE
MreB Thermatoga    182  MDEAIVQYVRETYR.............VAIG...ERTAERVKIEIGNVFPS..........KEND
Actin yeast        185  LTDYLMKILSERGY.............SFSTTAEREIVRDIKEKLCYVALDFEQEMQTAAQSSSI
Hsc70 bovine       233  FDNRMVNHFIAEFKRKHKKDISENKRAVRR...LRTACERAKRTLSS.................S
                         +                                           +  +

                                                                   S
                                                                   266
MreB Caulobacter   230  GLSIDVK..GRDLMQGVPREVRISEKQAADALAEPVG.........QIVEAVKVALEATPPELAS
MreB Thermatoga    221  ELETTVS..GIDLSTGLPRKLTLKGGEVREALRSVVV.........AIVESVRTTLEKTPPELVS
Actin yeast        237  EKSYELP..........DGQVITIGNERFRAPEALFHPSVLGLESAGIDQTTYNSIMKCDVDVRK
Hsc70 bovine       278  TQASIEIDSLYEG...IDFYTSITRARFEELNADLFR.........GTLDPVEKALRDAKL.DKS

                                                                 A/E T/P  C
                                                                   324    329
MreB Caulobacter   284  DIADKGIMLTGGGALLRGLDAEIRDHTG.........LPVTVADDPLSCVALGCGKVL.......
MreB Thermatoga    275  DIIERGIFLTGGGSLLRGLDTLLQKETG........ISVIRSEE.PLTAVAKGAGMVL...DKVN
Actin yeast        292  ELY.GNIVMSGGTTMFPGIAERMQKEITALAPSSMKVKIIAPPE.RKYSVWIGGSILASLTTFQQ
Hsc70 bovine       330  QIH..DIVLVGGSTRIPKIQKLLQDFFN........GKELNKSINPDEAVAYGAAVQA...AILS
                           hhhxGfxh-xxxxfxphhxpxh                pxxtthhGhhhxh
                                 Adenosine                         Connect II

MreB Caulobacter   333  .................EHPKWMKGVLESTLA
MreB Thermatoga    328  IL..............KKLQGAGgshhhhhh
Actin yeast        355  MWISKQEYDESGPSIVHHKCF...........
Hsc70 bovine       382  GDKse...........................
```

## Supplemental Figure 2

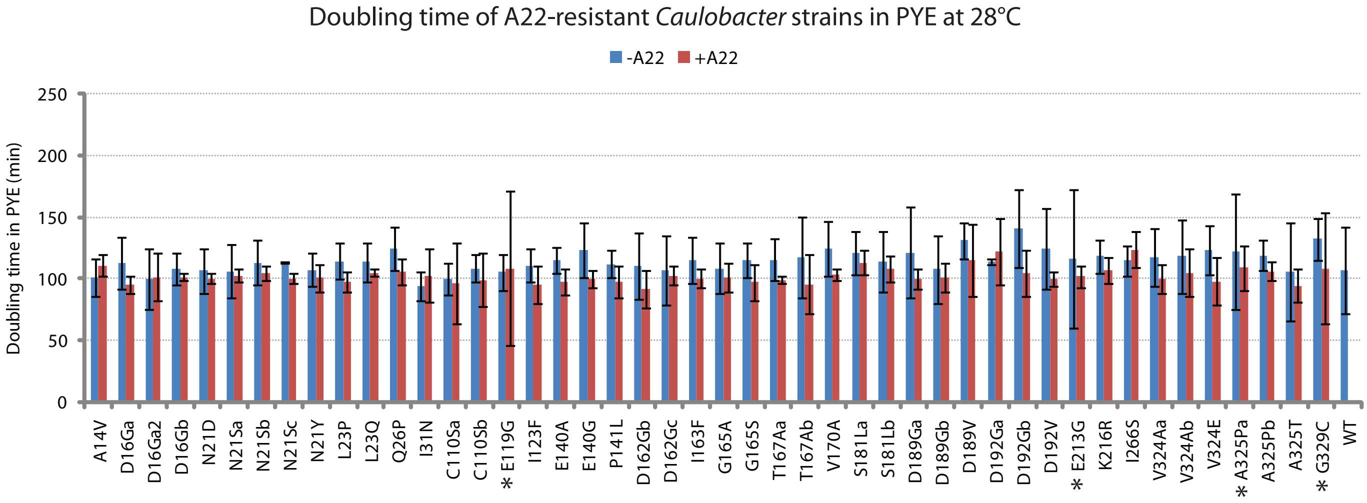

## Supplemental Figure 3

**A** Cell shapes in A22-resistant *Caulobacter* grown -A22

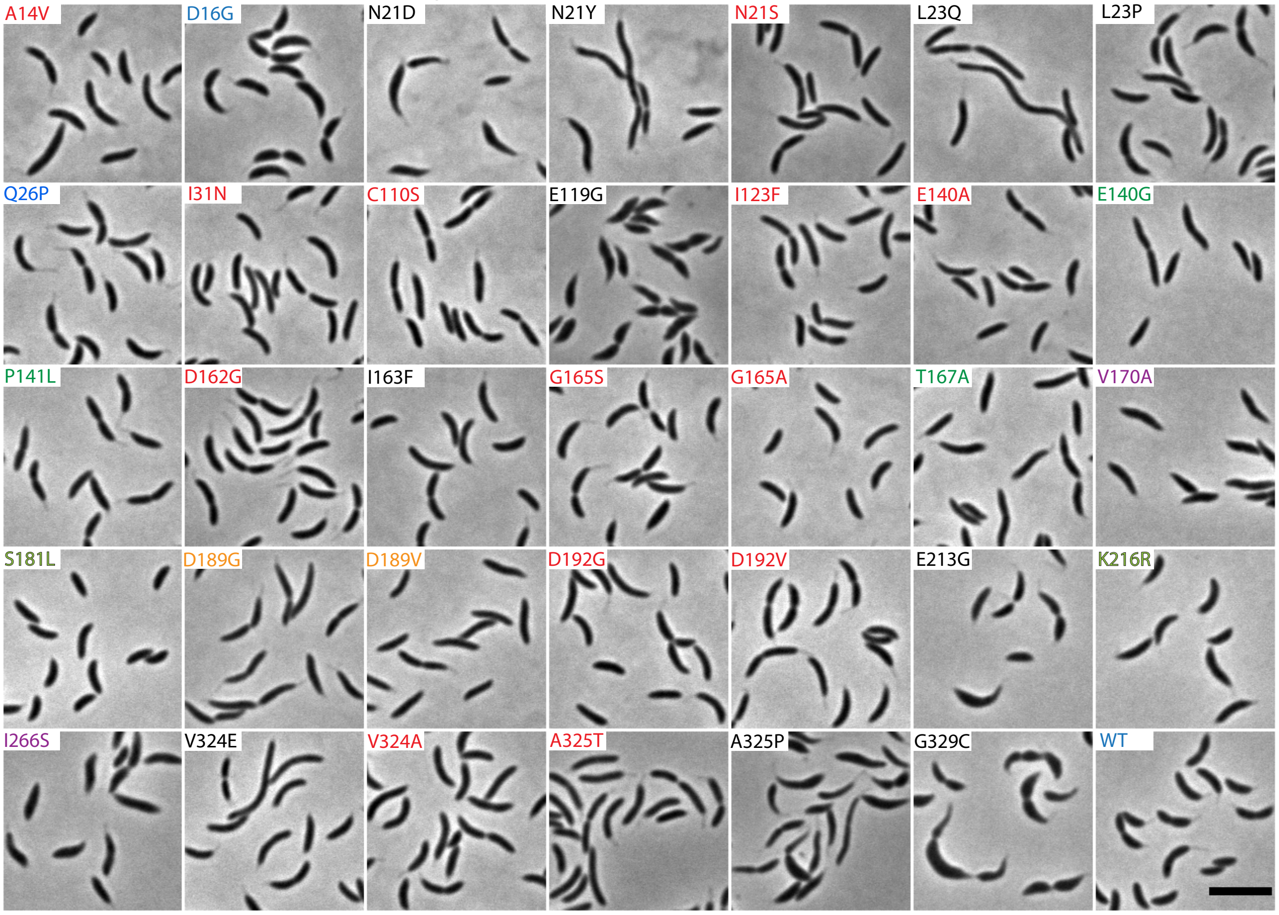


**B** Cell shapes in A22-resistant *Caulobacter* grown +A22

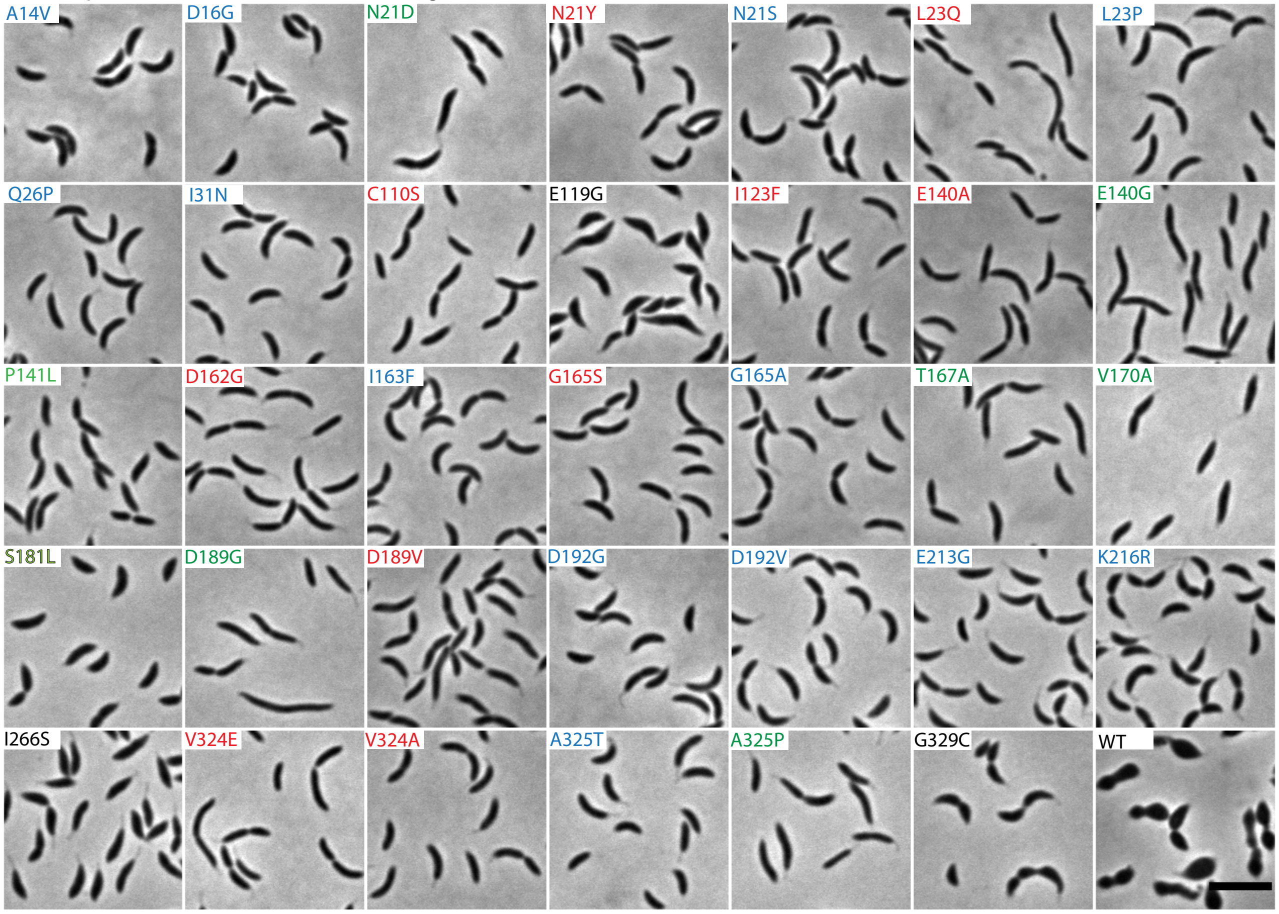

**Supplemental Figure 4**

Cell shapes of "mutation replicates" (independent isolates of the same mutation) grown -A22

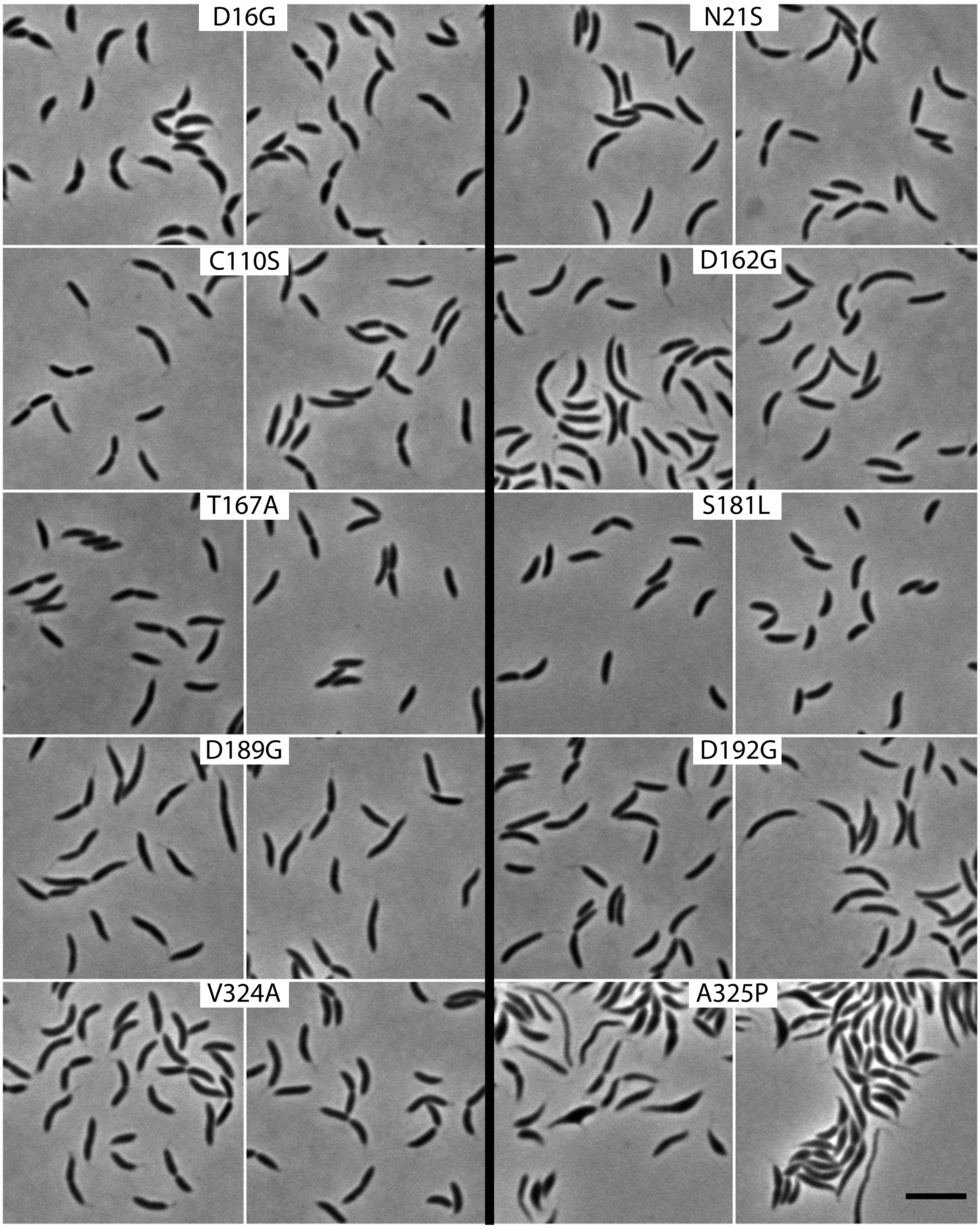

## Supplemental Figure 5

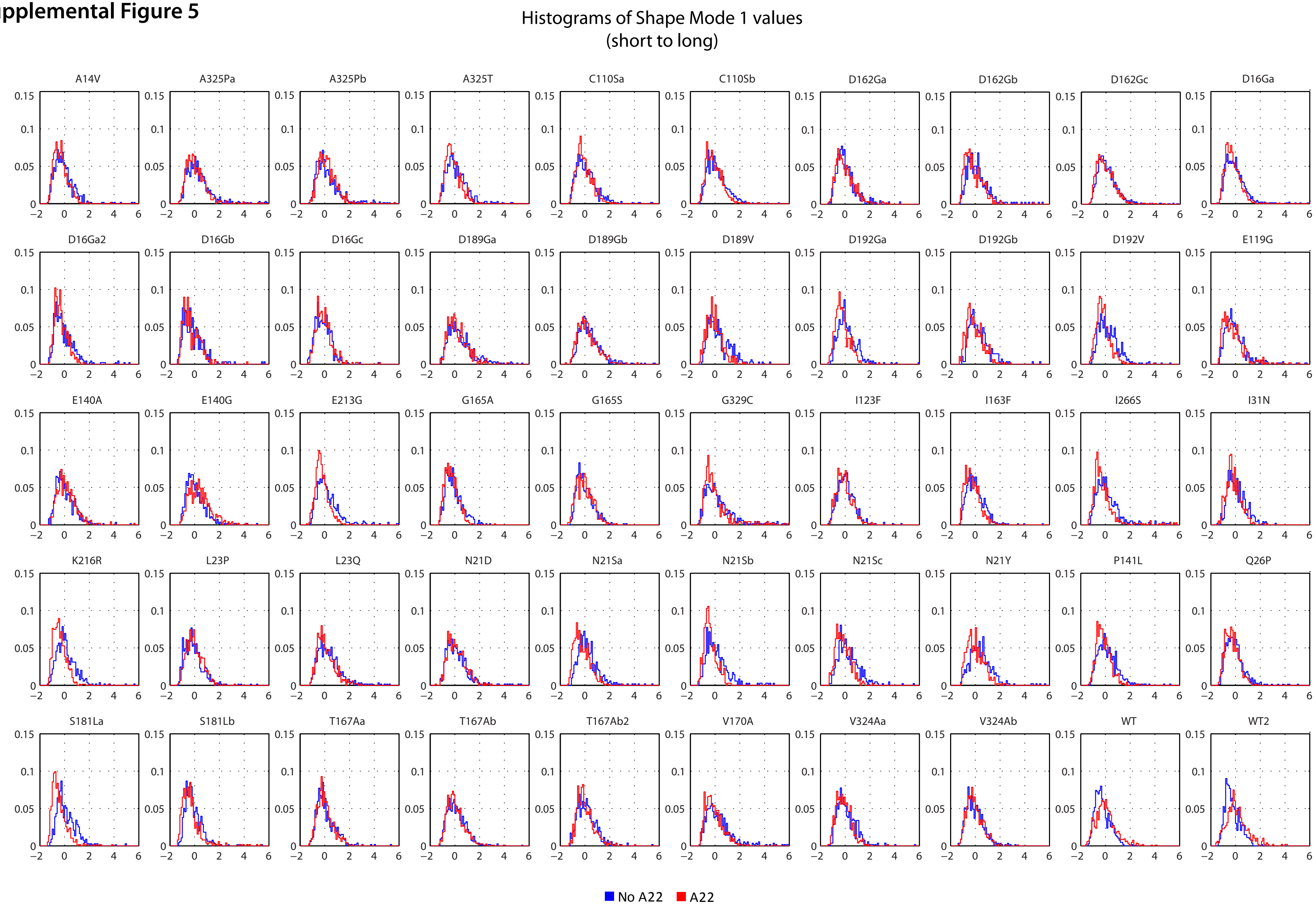

## Supplemental Figure 6

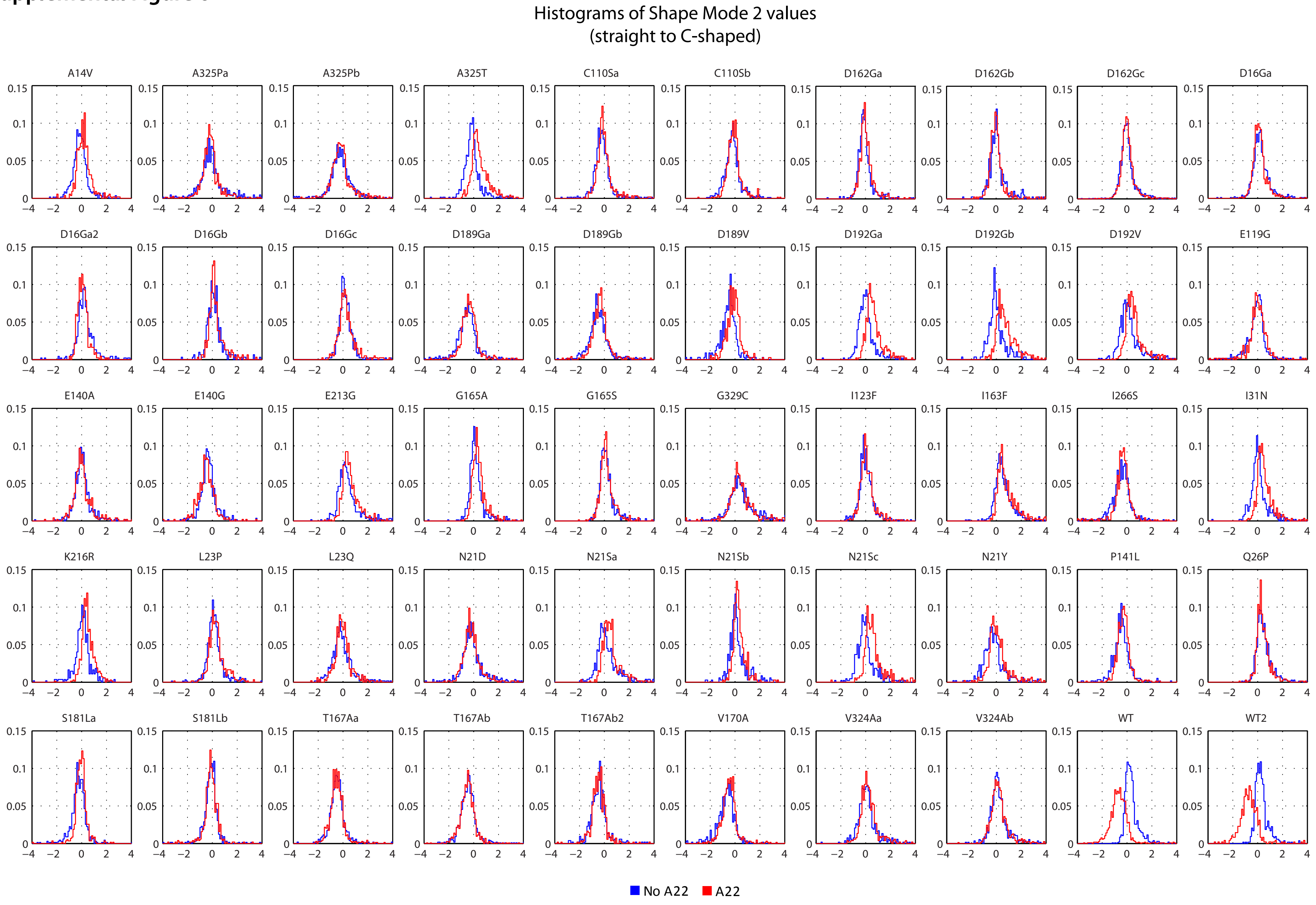

## Supplemental Figure 7

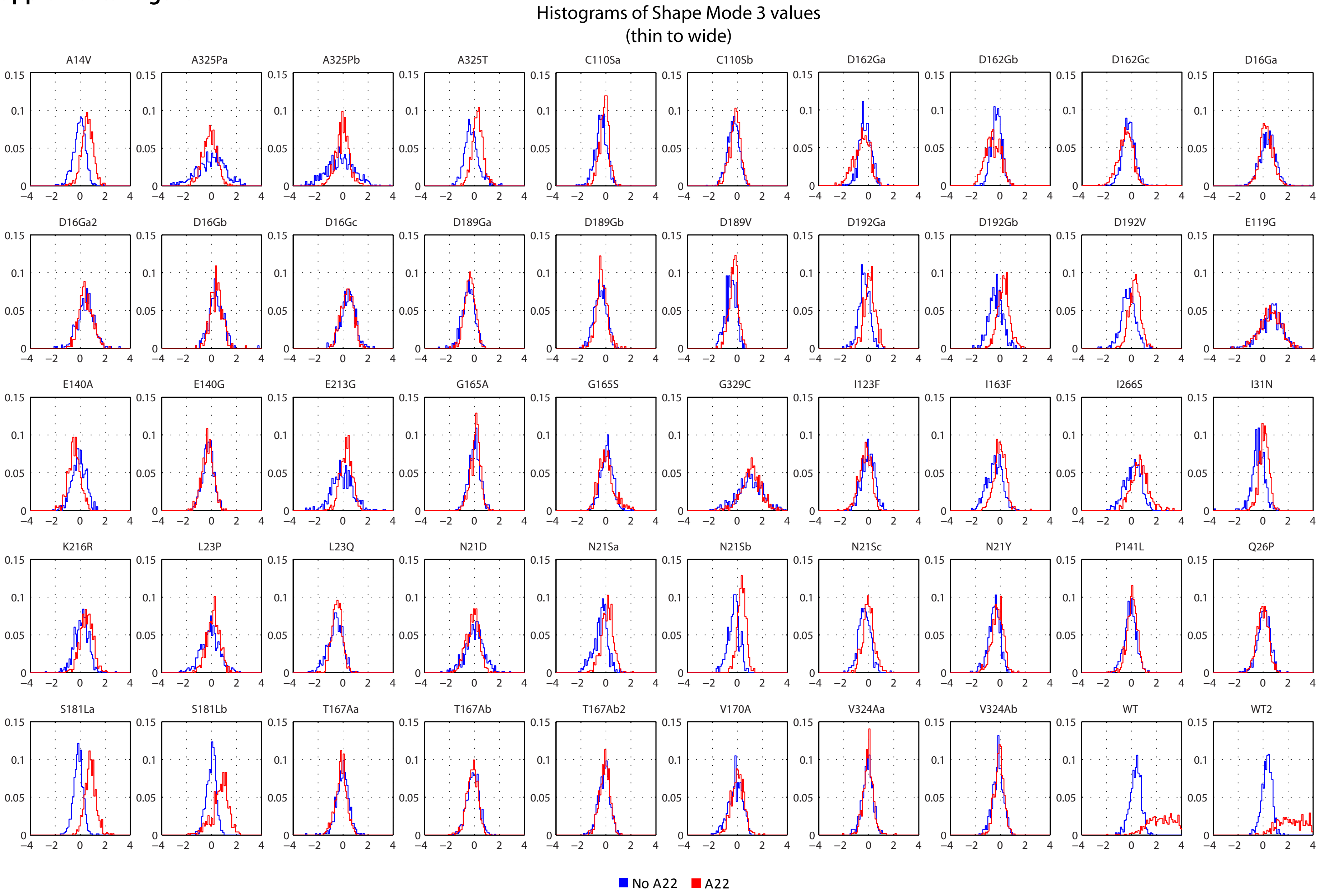

## Supplemental Figure 8

Histograms of absolute values of Shape Mode 4
(C-shaped to S-shaped)

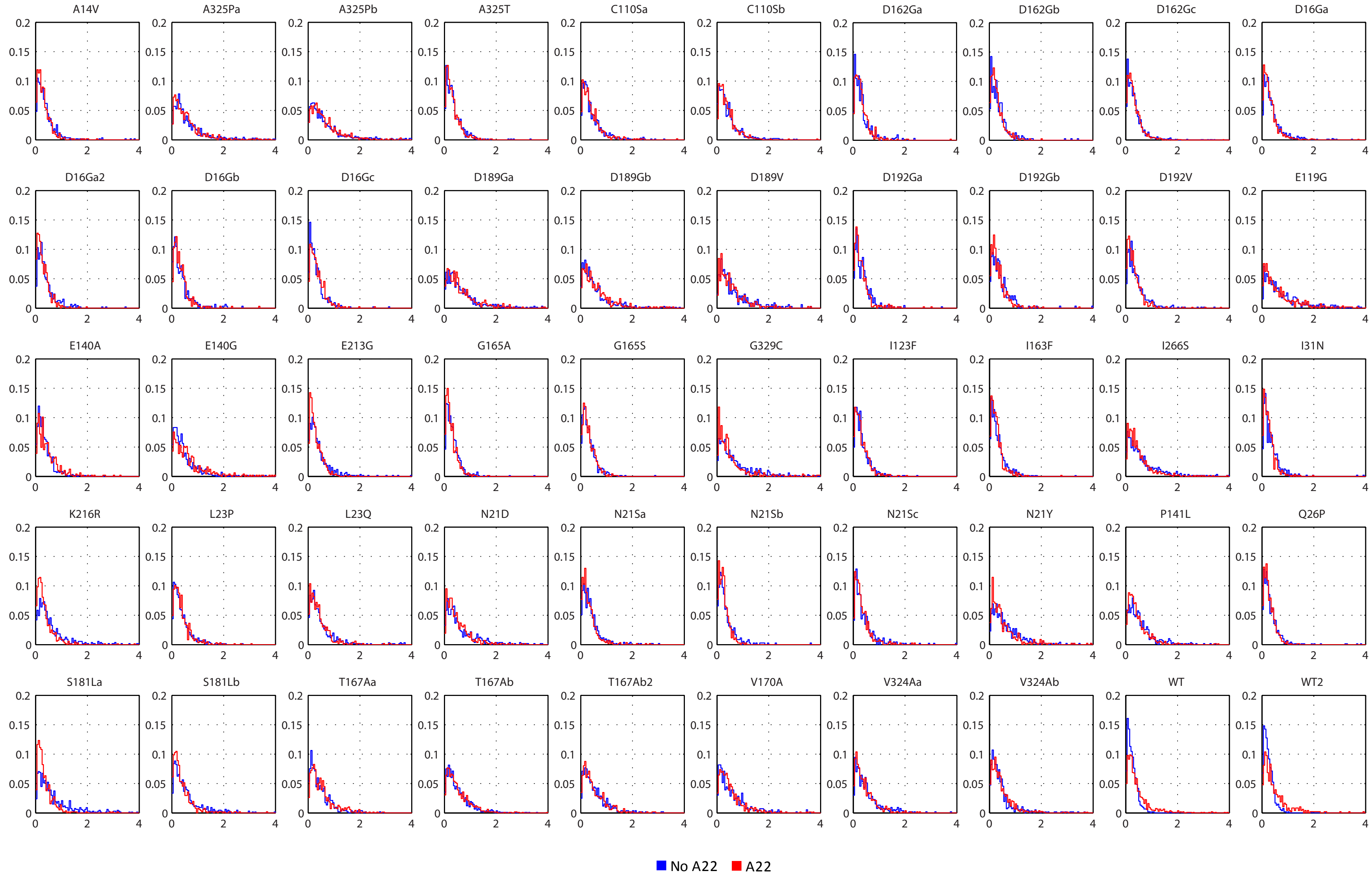

## Supplemental Figure 9

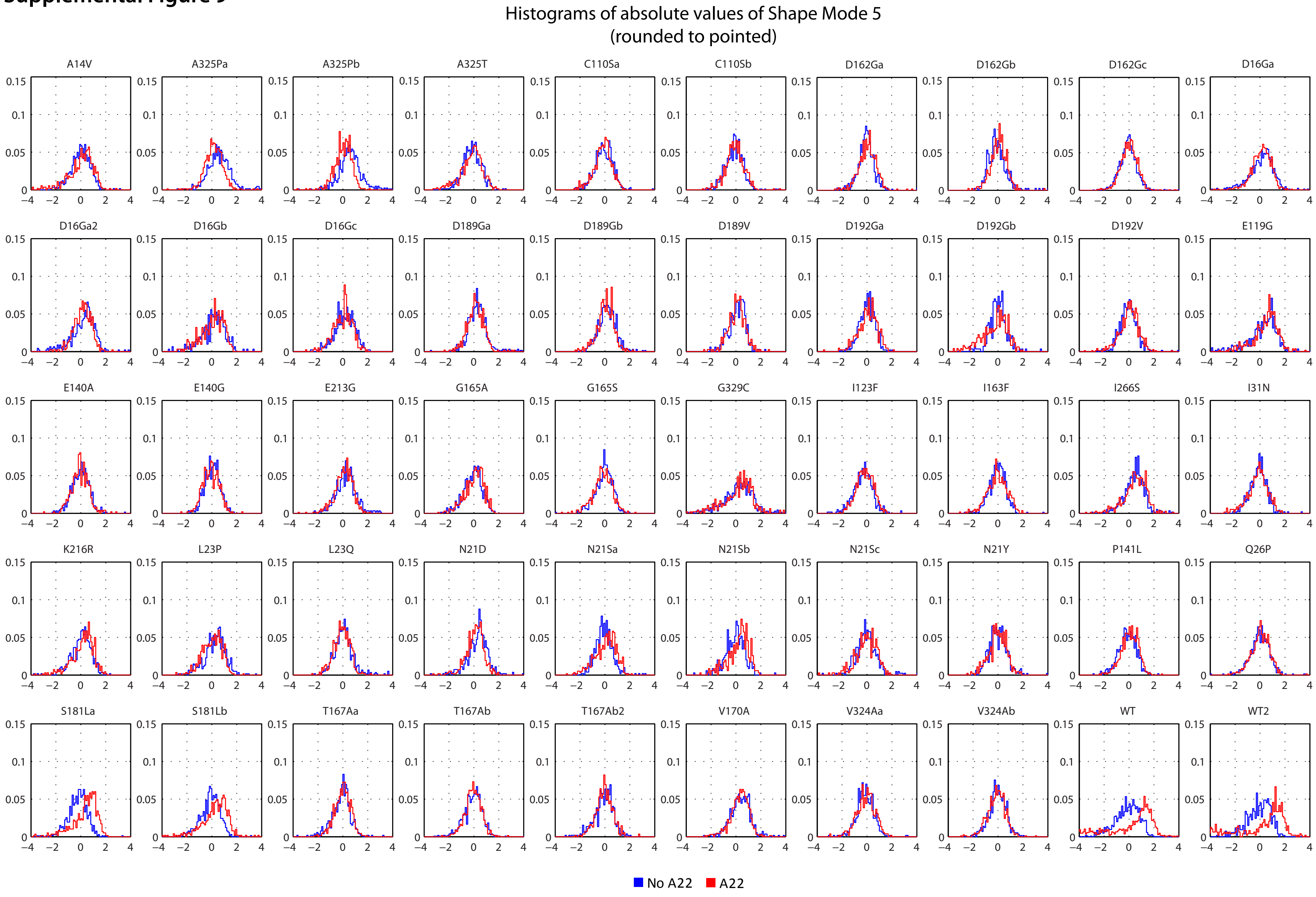

## Supplemental Figure 10

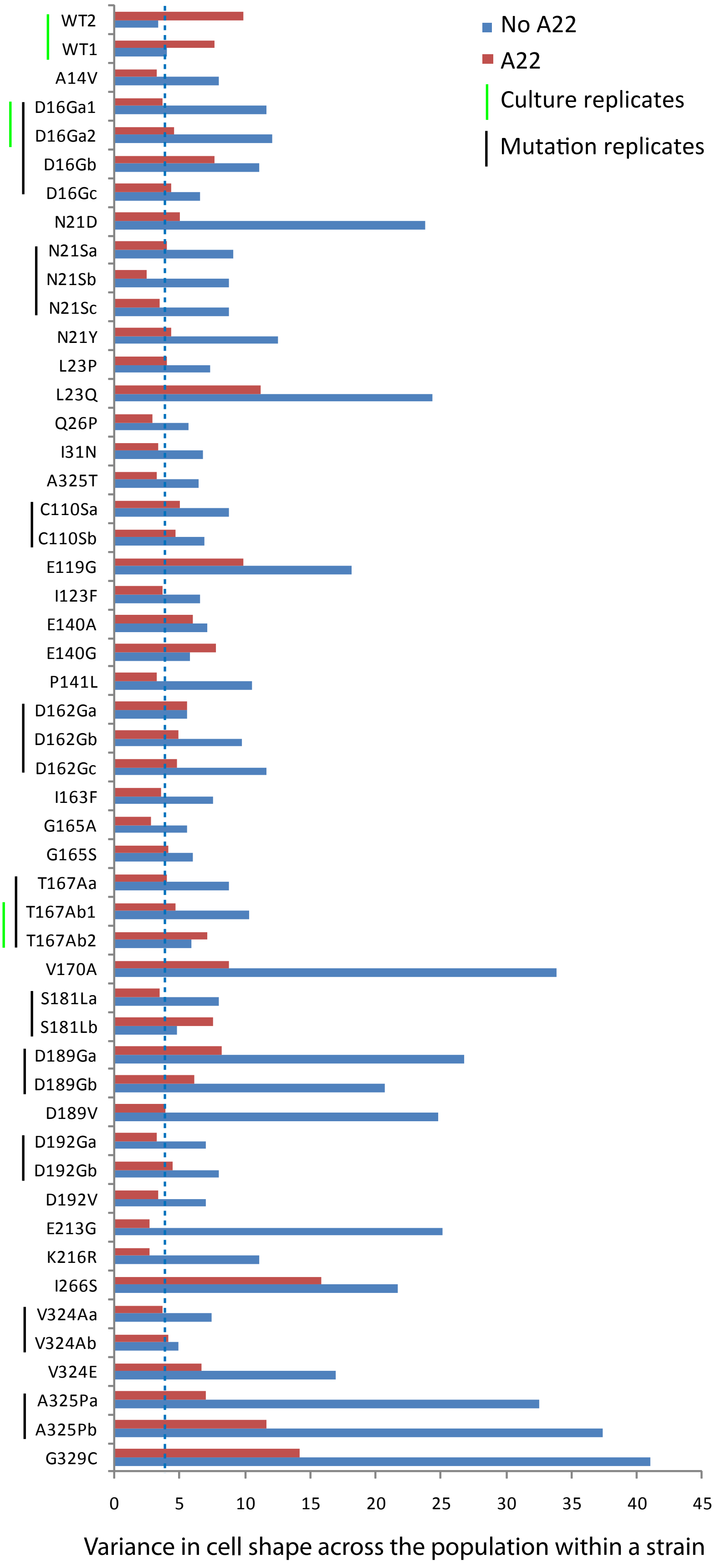

# Supplemental Figure 11

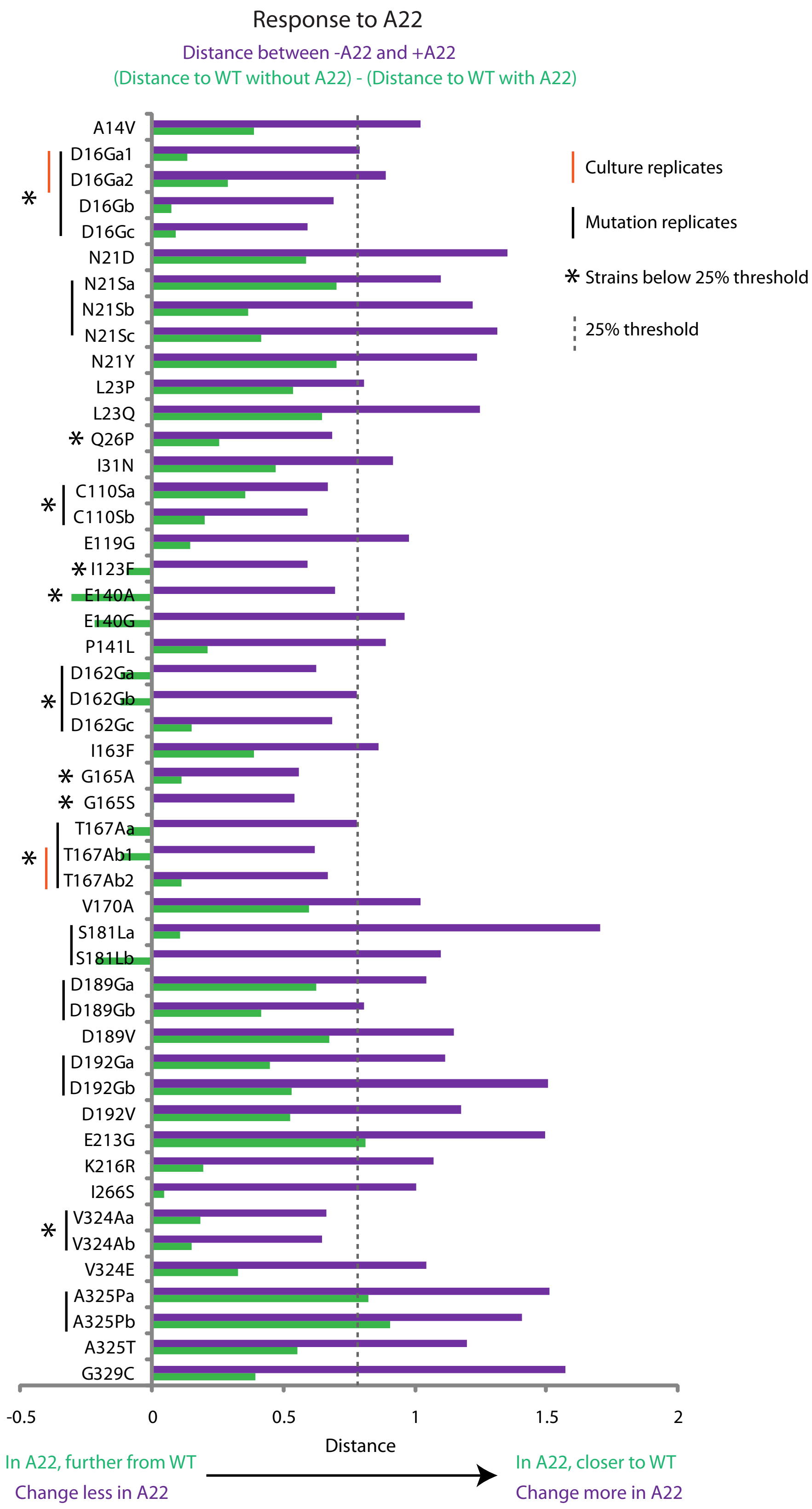

# Supplemental Figure 12

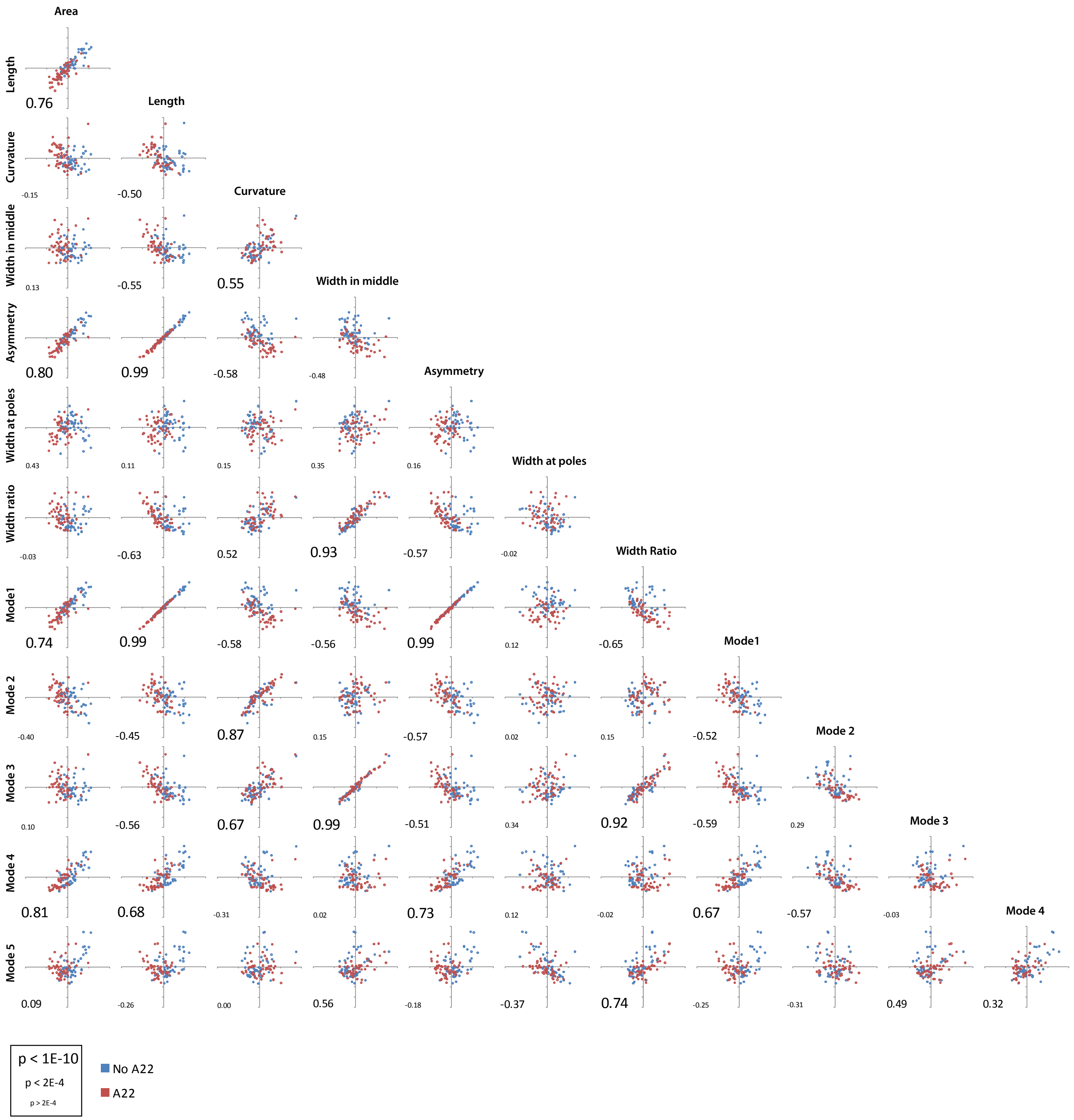

## Supplemental Figure 13

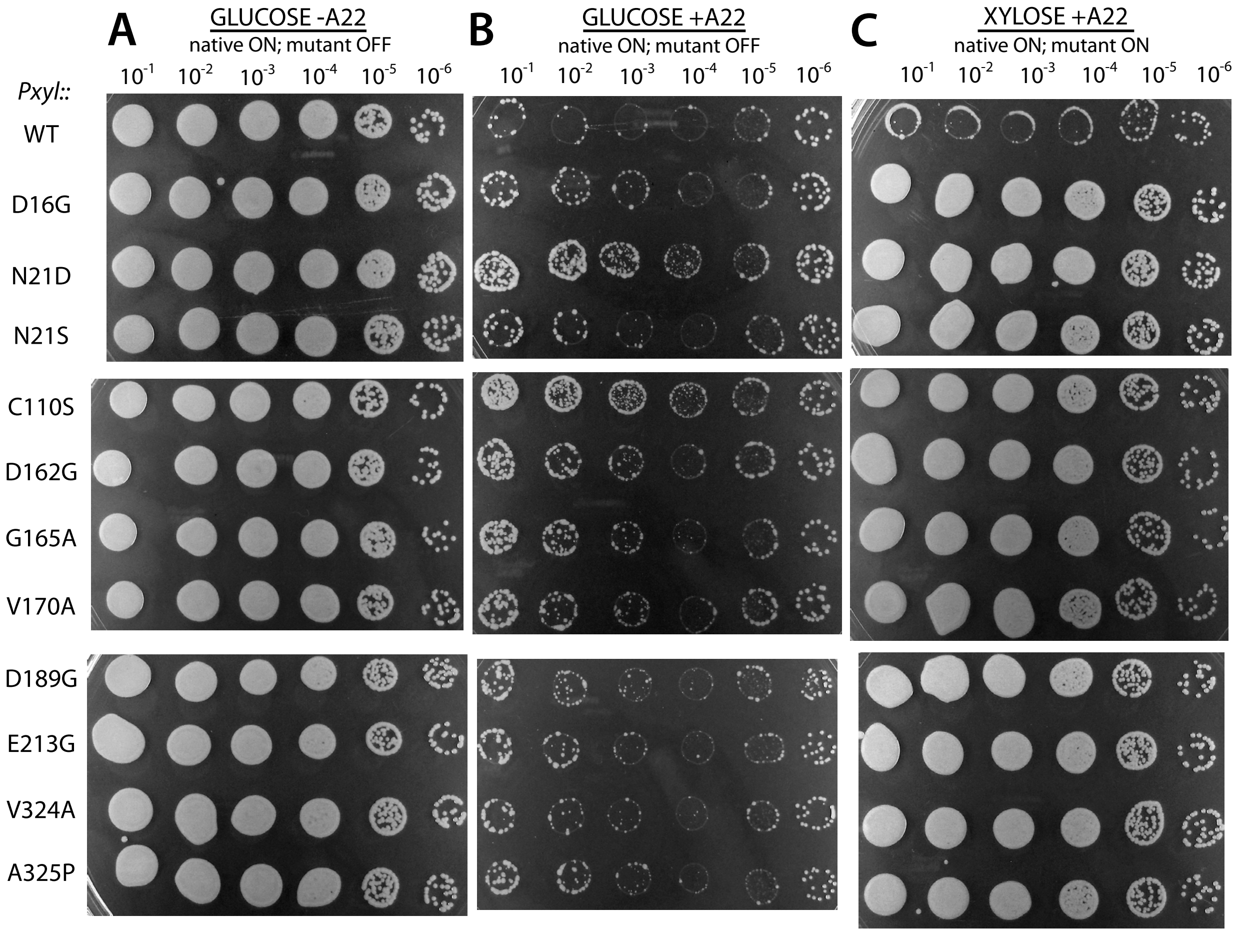


$P_{mreB}$::*mreB*$^{WT}$ + $P_{xyl}$::*venus-mreB*$^{mutant}$

## Supplemental Figure 14

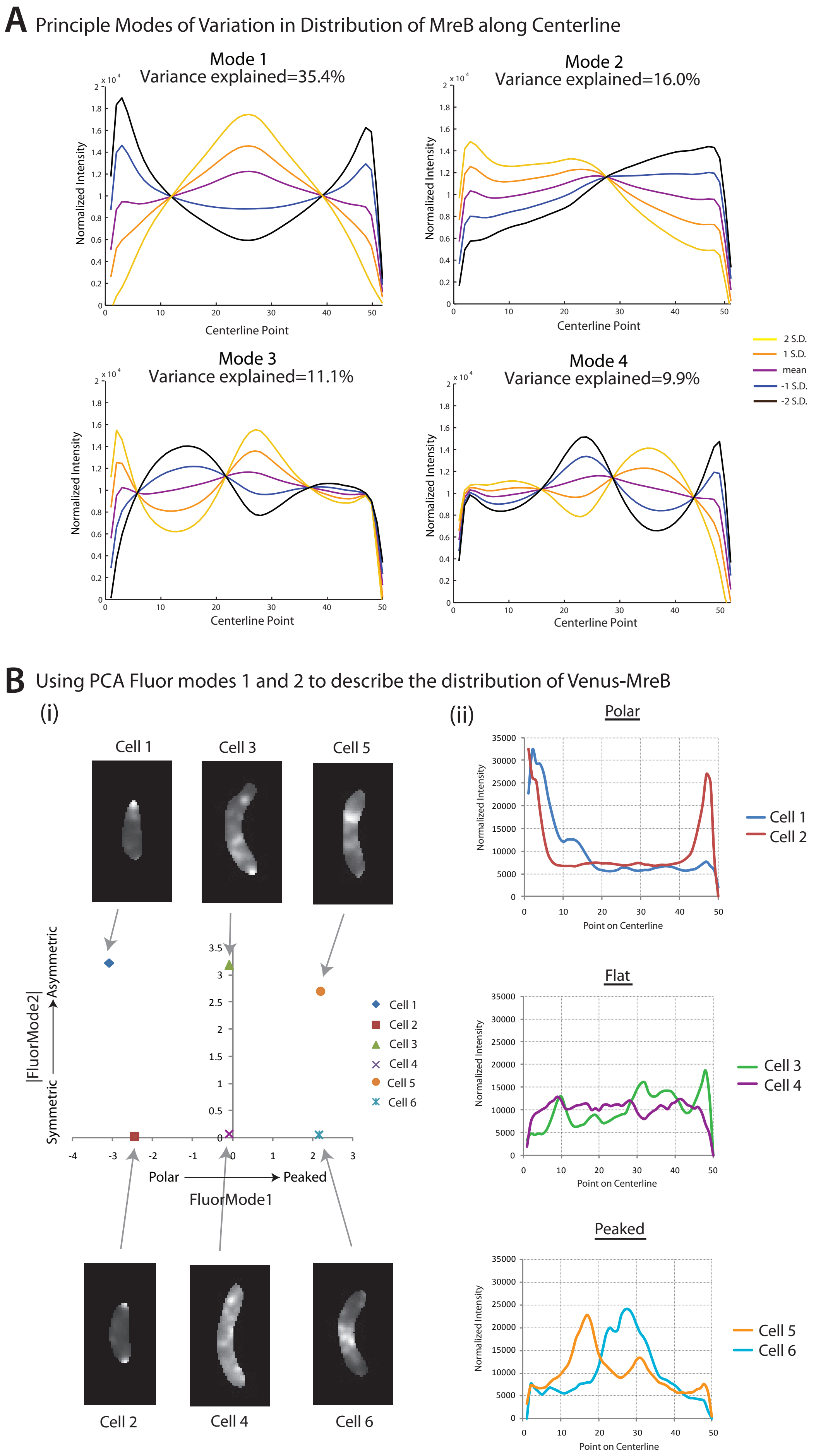

## Supplemental Figure 15

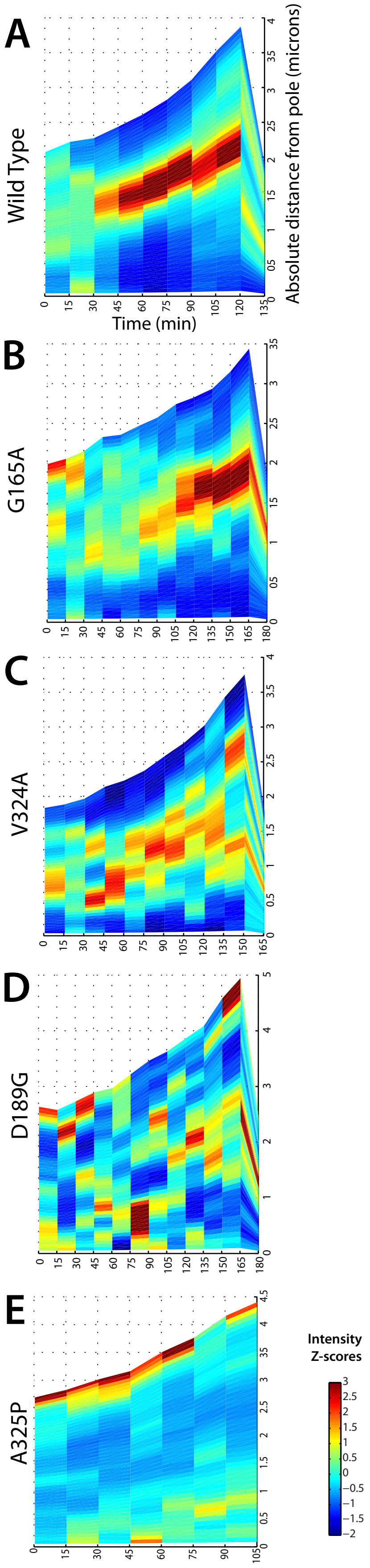

## Supplemental Figure 16

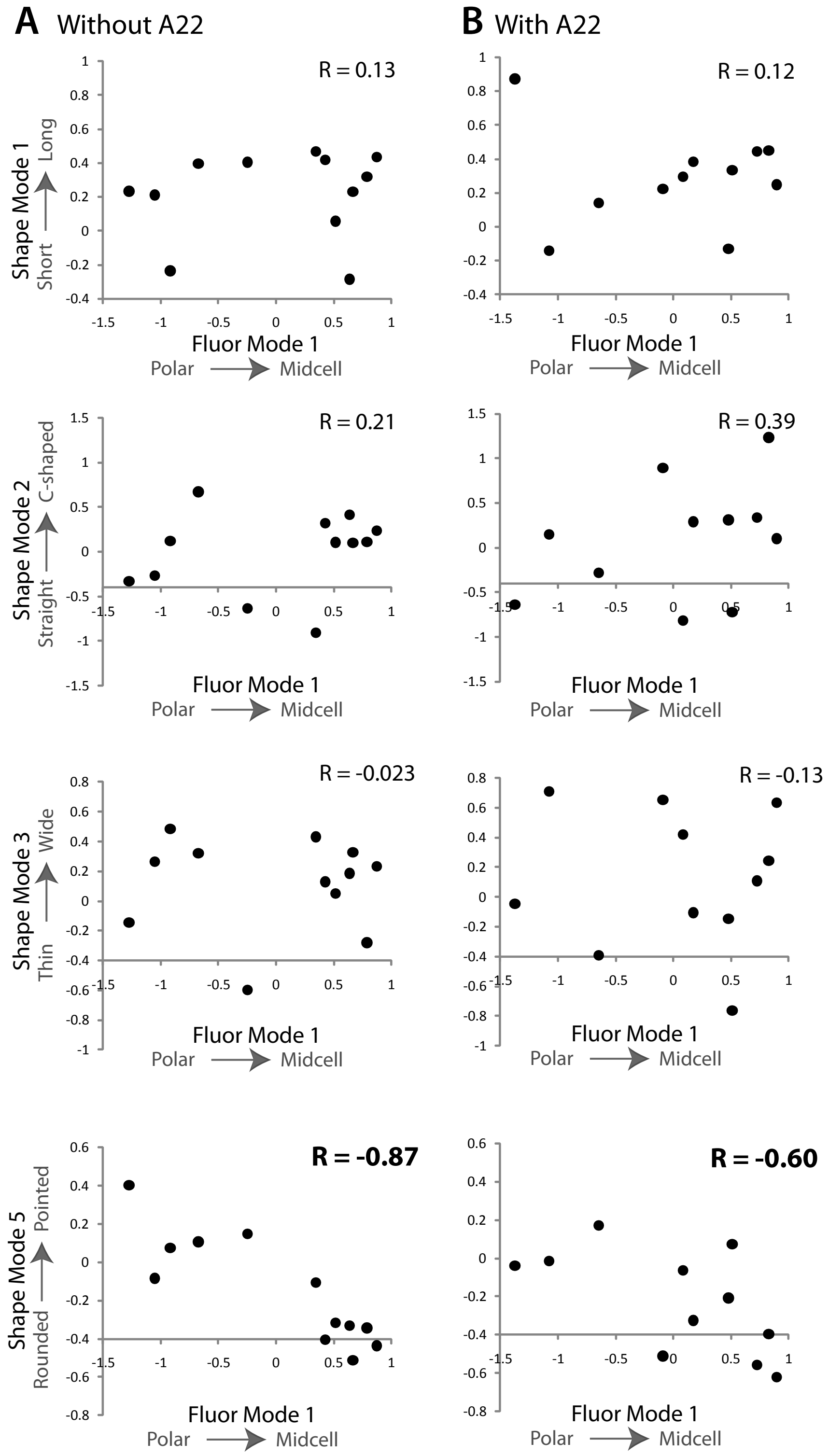

Supplement: Supplementary file 1 [file mmi0081-0368-SD1.pdf]
